# GLP-1 Receptor Agonists Plus Progestins and Endometrial Cancer Risk in Nonmalignant Uterine Diseases

**DOI:** 10.1001/jamanetworkopen.2025.58205

**Published:** 2026-02-10

**Authors:** Ting-Tai Yen, Tina Yi Jin Hsieh, Gin-Yi Lee, Eugene P. Toy, James Cheng-Chung Wei, Edward J. Tanner

**Affiliations:** 1 Department of Obstetrics and Gynecology, Texas Tech University Health Sciences Center El Paso, El Paso; 2 Department of Obstetrics and Gynecology, Beth Israel Deaconess Medical Center, Boston, Massachusetts; 3 Department of Medicine, Brigham and Women’s Hospital, Harvard Medical School, Boston, Massachusetts; 4 Division of Gynecologic Oncology, Department of Obstetrics and Gynecology, Texas Tech University Health Sciences Center El Paso, El Paso; 5 Department of Allergy, Immunology & Rheumatology, Chung Shan Medical University Hospital, Taichung, Taiwan; 6 Graduate Institute of Integrated Medicine, China Medical University, Taichung, Taiwan; 7 Institute of Medicine/Department of Nursing, Chung Shan Medical University, Taichung, Taiwan; 8 Office of Research and Development, Asia University, Taichung, Taiwan; 9 Division of Gynecologic Oncology, Department of Obstetrics and Gynecology, Johns Hopkins School of Medicine, Baltimore, Maryland

## Abstract

**Importance:**

As endometrial cancer (EC) incidence rises, particularly among individuals with obesity and metabolic disorders, effective strategies targeting hormonal and metabolic risks are needed.

**Objective:**

To evaluate EC risk in patients with endometrial hyperplasia (EH) or benign uterine pathology treated with progestins vs combined progestins and glucagon-like peptide-1 receptor agonists (GLP-1RAs).

**Design, Setting, and Participants:**

This cohort study used TriNetX to analyze EC and hysterectomy among adult women with EH or benign uterine pathology who received progestins between May 1, 2005 (GLP-1RA approval date), and December 31, 2022. Analyses were based on deidentified electronic health records identified via *International Statistical Classification of Diseases and Related Health Problems, Tenth Revision* codes, and data were obtained on February 23, 2025. Four treatment comparisons were analyzed: GLP-1RA plus progestins vs progestins only; GLP-1RA plus progestins vs metformin progestins; triple therapy (GLP-1RA, metformin, and progestins) vs metformin plus progestins; triple therapy vs progestins only. Subgroup analyses GLP-1RA plus progestins vs progestins only stratified patients by progestin route, risk level, body mass index (BMI), and age.

**Exposures:**

GLP-1RAs, progestins, and/or metformin.

**Main outcomes and measures:**

The primary outcome was the incidence of EC. The secondary outcome was the incidence of subsequent hysterectomy.

**Results:**

A total of 18 414 and 426 406 adult female patients received GLP-1RA combined with progestin and progestin alone, respectively (mean [SD] age, 43.1 [10.2] years vs 35.2 [10.9] years). GLP-1RA with progestins was associated with a significantly lower risk of EC compared with progestins only (HR, 0.34 [95% CI, 0.27-0.44]). This protective association remained consistent across subgroups, stratified by progestin route, baseline risk, BMI, and age. GLP-1RA plus progestins also showed a lower EC risk than metformin plus progestins (HR, 0.30 [95% CI, 0.15-0.59]). Triple therapy was more effective in reducing EC risk than dual (metformin plus progestins) (HR, 0.37 [95% CI, 0.25-0.53]) or progestin monotherapy (HR, 0.44 [95% CI, 0.29-0.66]). Hysterectomy rates were lower in the GLP-1RA plus progestins group at 2-year (HR, 0.47 [95% CI, 0.42-0.53]) and 5-year (HR, 0.59 [95% CI, 0.54-0.64]) follow-up.

**Conclusions and Relevance:**

In this cohort study of women with benign uterine pathology or endometrial hyperplasia, combined GLP-1RA and progestin was associated with reduced EC risk. Further investigation is warranted to assess its applicability and underlying mechanisms.

## Key Points

**Question:**

Do GLP-1 receptor agonists enhance the effectiveness of progestins in reducing endometrial cancer (EC) risk among patients with nonmalignant uterine conditions?

**Findings:**

In this cohort study with 444 820 participants, combined GLP-1RA with progestin was associated with a significantly lower risk of developing EC compared with progestin alone, across subgroups stratified by body mass index, age, baseline risk level, and progestin route.

**Meaning:**

These results suggest that adding GLP-1RAs to progestin therapy in women with benign uterine diseases or hyperplasia may be associated with reduced EC risk.

## Teaser

This cohort study compares the associations of glucagon-like peptide-1 (GLP-1) receptor agonists and of progestins with risk of endometrial cancer among US women with endometrial hyperplasia or other benign uterine pathologies.

## Introduction

Endometrial cancer (EC) is the most common gynecologic malignant neoplasm in developed countries, with an estimated 69 120 new cases expected in 2025.^1^ Approximately 90% of patients with EC present with abnormal uterine bleeding (AUB),^2^ creating an opportunity for earlier detection and prevention within AUB populations. Historically, the risks of EC and its precursor, endometrial intraepithelial neoplasia (EIN), among patients with AUB were thought to be low (0.3% to 1.3%).^3^ Recently, studies report higher risks when metabolic factors are considered.^4,5,6^ Obesity, insulin resistance, type 2 diabetes (T2D), and unopposed estrogen are key drivers of EC through endometrial proliferation and carcinogenesis.^7,8,9,10^

Progestin therapy is the cornerstone of nonsurgical management for abnormal uterine bleeding and endometrial hyperplasia (EH). Options include megestrol acetate (MA), medroxyprogesterone acetate (MPA), and levonorgestrel-releasing intrauterine devices (LNG-IUD). In addition, metabolic risk optimization, such as weight loss and glucose control, may reduce EC risk.^11^ By mitigating the effects of unopposed estrogen-driven endometrial proliferation, progestins have been shown to achieve higher rates of disease regression.^11,12^ Patients receiving progestin therapy may therefore benefit from adjunctive treatments that target these metabolic factors, potentially enhancing the effectiveness of progestin therapy.

Glucagon-like peptide-1 receptor agonists (GLP-1RAs) have been Food and Drug Administration (FDA)–approved for the treatment of T2D since 2005 and for weight loss since 2014.^13,14^ Beyond their metabolic effects, emerging evidence suggests that GLP-1RAs may possess broader therapeutic potential in several obesity-related malignant neoplasms,^15^ including antitumorigenic properties mediated through multiple signaling pathways in different organ systems.^16^ Notably, GLP-1 receptors are expressed in both malignant and nonmalignant endometrial tissues,^17^ and preclinical models have demonstrated that treatment with GLP-1RAs combined with progestins can significantly reduce tumor cell viability of progesterone receptor (PR) tumors, across both high-expression and low-expressing tumors.^17^

However, data on the impact of GLP-1RA combined with progestins on risk of developing EC compared with progestin alone or other metabolic therapies such metformin in patients with precancer or without cancer remain limited. Therefore, we conducted a retrospective cohort study utilizing a large clinical database to evaluate whether the use of GLP-1RAs in combination with progestin therapy is associated with a reduced risk of developing EC among women with endometrial hyperplasia (EH) or other benign uterine pathologies.

## Methods

### Database

Data were obtained from TriNetX, a global federated health research network that provides real-time access to deidentified electronic health records (EHRs) from large health care organization.^18,19^ The primary analysis used the Global Collaborative Network (142 health care organizations [HCOs]), and validation was performed using the US Collaborative Network (68 HCOs), based on data available as of February 23, 2025 (eMethods in Supplement 1). The study was approved by the institutional review board of Chung-Shan Medical University, Taiwan. Informed consent was waived in accordance with institutional review board policy because the study used deidentified data. This retrospective study complied with the Strengthening the Reporting of Observational Studies in Epidemiology (STROBE) reporting guideline.

### Study Population

Eligibility criteria included women aged 18 years or older who were diagnosed with EH or benign uterine pathology and received progestin from May 1, 2005 (GLP-1RA approval date), to December 31, 2022. Benign uterine pathology included abnormal uterine bleeding (AUB), submucosal leiomyoma, endometrial polyp, or simple hyperplasia, and was classified as the low-risk group. EH was classified as the high-risk group (*International Statistical Classification of Diseases and Related Health Problems, Tenth Revision *[*ICD-10*] codes N85.0, N85.00, or N85.02), which includes unspecified EH and EIN. All women in this study received progestins, which included either a LNG-IUD or systemic progestins such as MA or MPA. In addition to progestins, some participants also received GLP-1RA, metformin, or both GLP-1RA and metformin. These medications were not necessarily prescribed for the treatment of EH or benign uterine pathology. The index date was defined as the first date on which all medications required for the assigned group were initiated. All study participants were followed up from the index event (start day of treatment) to the occurrence of EC, at least 2-year follow-up, death, or loss follow-up, whichever occurred first. Exclusion criteria included women who had hysterectomy or EC diagnosis prior to the index event. The primary outcome was the risk of EC. The secondary outcome was hysterectomy incidence.

### Study Design

**Figure 1.**
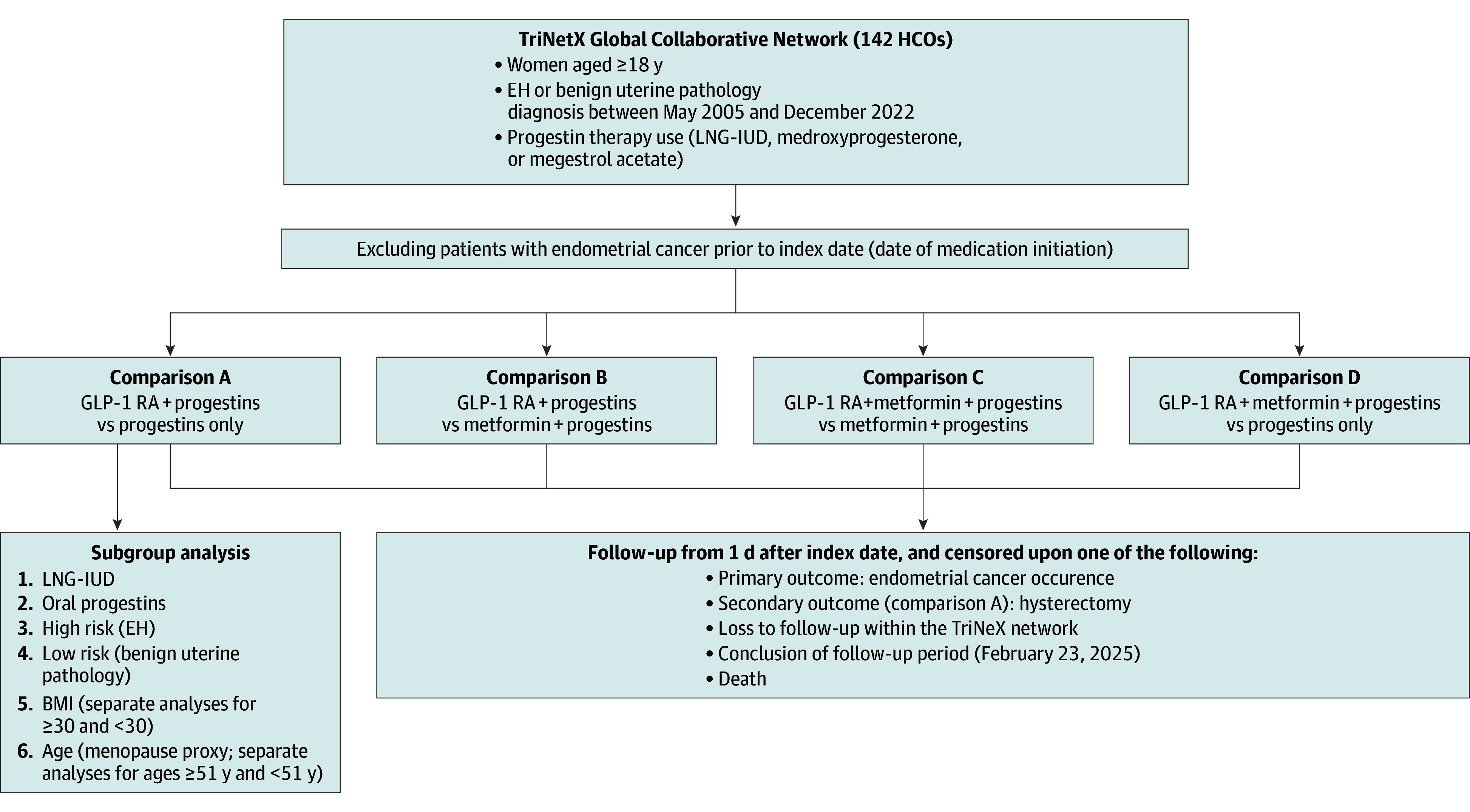
Flow Diagram of Study Design Primary outcomes were compared between treatment groups across 4 comparisons (A through D). Subgroup analyses and secondary outcomes were evaluated within comparison A. BMI indicates body mass index (calculated as weight in kilograms divided by height in meters squared); EH, endometrial hyperplasia; GLP-1RA, glucagon-like peptide 1 receptor agonist; HCO, health care organizations; LNG-IUD, levonorgestrel-releasing intrauterine devices.

We defined 4 main comparisons for the primary outcomes (risks of EC) between treatment groups (Figure 1). Comparison A was defined as GLP-1RA plus progestin vs progestin only; comparison B, GLP-1RA plus progestin vs metformin plus progestin; comparison C, triple therapy of GLP-1RA, metformin, and progestin vs metformin plus progestin; and comparison D, triple therapy of GLP-1RA, metformin, and progestin vs progestin only. We validated the analysis by repeating the comparison A using the US Collaborative Network.

Given the heterogeneity of the study population, we further performed subgroup analysis within Comparison A (GLP-1RA plus progestin vs progestin only) to identify potential effect modifiers and assess the generalizability of the findings. We stratified the comparison A population based on different variables, including progestin route (LNG-IUD or systemic progestins), risk level (high or low), body mass index (BMI; calculated as weight in kilograms divided by height in meters squared), and age. The BMI subanalysis categorized patients into 2 groups: obese (BMI 30 or above) and nonobese (BMI below 30). The age subanalysis stratified patients into menopausal (51 years or older) and nonmenopausal (younger than 51 years) groups, using age of 51 years as a proxy indicator for menopause.^20^ The secondary outcome analysis was performed in comparison A (GLP-1RA plus progestin vs progestin only), evaluating the incidence of subsequent total hysterectomy at 2 and 5 years after the date of index event. Patient characteristics were retrieved from TriNetX using the comorbidities, diagnostic, procedural, and medication codes recorded within one year prior to the index event (eMethods and eTable 1 in the Supplement 1).

### Statistical Analysis

The analyses were performed using the TriNetX analytic platform. Propensity score matching (PSM) of 1:1 was used to match patients within each comparison (covariates defined in eTable 1 in Supplement 1). A standardized mean difference (SMD) threshold of 0.1 was used to assess covariate balance and effectiveness of PSM. Kaplan-Meier survival analysis and Cox proportional hazard models were performed to estimate hazard ratios (HRs) with 95% CIs.

## Results

The study population included total of 444 820 women (mean [SD] age, 35.5 [11.0] years). By race and ethnicity, 93 758 identified as Black (21.1%), 57 104 Hispanic or Latino (12.8%), and 247 597 White (55.7%).

### Endometrial Cancer Risk in Patients Using GLP-1RA Plus Progestins Compared With Progestins Only (Comparison A)

A total of 18 414 patients who received GLP-1RA combined with progestins (GLP-1RA plus progestins) and 426 406 patients who received progestins alone were identified (Table 1). The mean (SD) age at index was 43.1 (10.2) years in the GLP-1RA plus progestins group and 35.2 (10.9) years in the progestins only group. The majority of patients in both groups were White, accounting for 10 083 (54.8%) in the GLP-1RA plus progestins group and 237 514 (55.7%) in the progestins only group. Most patients were non-Hispanic, with 13 916 (75.6%) in the GLP-1RA plus progestins group and 291 880 (68.5%) in the progestins group.

**Table 1.** Patient Characteristics in Comparison A (GLP-1RA Plus Progestins vs Progestins Only) Before and After Propensity Score Matching

Characteristics^a^	Before PSM			After PSM, patients, No. (%)		
	GLP-1RA + progestins, patients, No. (%) (n = 18 414)	Progestins only, patients, No. (%) (n = 426 406)	SMD^b^	GLP-1RA + progestins, patients, No. (%) (n = 15 747)	Progestins only, patients, No. (%) (n = 15 747)	SMD^b^
Age at index, mean (SD), y	43.1 (10.2)	35.2 (10.9)	0.746	42.4 (10.0)	43.2 (12.0)	0.067
Race						
American Indian or Alaska Native	113 (0.6%)	1622 (0.4)	0.033	97 (0.6%)	72 (0.5%)	0.022
Asian	537 (2.9)	25 666 (6.0)	0.151	471 (3.0)	410 (2.6)	0.023
Black or African American	5433 (29.5)	88 325 (20.7)	0.204	4545 (28.9)	4576 (29.1)	0.004
Pacific Islander^c^	181 (1.0)	4119 (1.0)	0.002	152 (1.0)	184 (1.2)	0.02
White	10 083 (54.8)	237 514 (55.7)	0.019	8684 (55.1)	8782 (55.8)	0.013
Other^d^	677 (3.7)	18 037 (4.2)	0.028	582 (3.7)	572 (3.6)	0.003
Unknown race	1390 (7.5)	51 123 (12.0)	0.15	1216 (7.7)	1151 (7.3)	0.016
Ethnicity						
Hispanic or Latino	2270 (12.3)	54 834 (12.9)	0.016	1963 (12.5)	1965 (12.5)	<0.001
Not Hispanic or Latino	13 916 (75.6)	291 880 (68.5)	0.159	11 789 (74.9)	11 862 (75.3)	0.011
Unknown ethnicity	2228 (12.1)	79 692 (18.7)	0.183	1995 (12.7)	1920 (12.2)	0.014
Comorbidities						
Socioeconomic risks^e^	501 (2.7)	9023 (2.1)	0.039	378 (2.4)	378 (2.4)	<0.001
Nicotine dependence	1373 (7.5)	23 384 (5.5)	0.08	1132 (7.2)	1070 (6.8)	0.015
Hypertensive diseases	8626 (46.8)	42 791 (10.0)	0.894	6573 (41.7)	6808 (43.2)	0.03
T2D	8547 (46.4)	13 890 (3.3)	1.153	6013 (38.2)	5729 (36.4)	0.037
T2D with complications	4332 (23.5)	3970 (0.9)	0.735	2713 (17.2)	2265 (14.4)	0.078
Cerebrovascular diseases	357 (1.9)	3267 (0.8)	0.102	302 (1.9)	328 (2.1)	0.012
Liver diseases	1711 (9.3)	7177 (1.7)	0.339	1236 (7.8)	1198 (7.6)	0.009
Hyperlipidemia	4446 (24.1)	15 561 (3.6)	0.62	3200 (20.3)	3181 (20.2)	0.003
Acute myocardial infacrction	165 (0.9)	910 (0.2)	0.092	134 (0.9)	140 (0.9)	0.004
Heart failure	718 (3.9)	3153 (0.7)	0.211	551 (3.5)	588 (3.7)	0.013
Atherosclerosis	143 (0.8)	678 (0.2)	0.091	117 (0.7)	122 (0.8)	0.004
Peripheral vascular disease	172 (0.9)	702 (0.2)	0.104	132 (0.8)	136 (0.9)	0.003
CKD	878 (4.8)	4541 (1.1)	0.221	690 (4.4)	768 (4.9)	0.024
Gastric ulcer	92 (0.5)	1022 (0.2)	0.043	79 (0.5)	81 (0.5)	0.002
Connective tissue disorders	407 (2.2)	4571 (1.1)	0.09	318 (2.0)	361 (2.3)	0.019
HIV disease	102 (0.6)	1244 (0.3)	0.04	82 (0.5)	67 (0.4)	0.014
Chronic lower respiratory diseases	3787 (20.6)	35 089 (8.2)	0.357	2963 (18.8)	3095 (19.7)	0.021
Pregnancy	241 (1.3)	31 292 (7.3)	0.3	231 (1.5)	228 (1.4)	0.002
Service types						
Office or outpatient Services	13 573 (73.7)	207 443 (48.6)	0.532	11 287 (71.7)	11 424 (72.5)	0.019
Preventive medicine services	5591 (30.4)	82 282 (19.3)	0.258	4677 (29.7)	4699 (29.8)	0.003
ED services	4595 (25.0)	74 237 (17.4)	0.185	3750 (23.8)	3760 (23.9)	0.001
Hospital inpatient services	1283 (7.0)	22 633 (5.3)	0.069	1059 (6.7)	1110 (7.0)	0.013
Medications						
Metformin	7210 (39.2)	10 025 (2.4)	1.018	4975 (31.6)	4721 (30.0)	0.035
Insulins	3979 (21.6)	8494 (2.0)	0.638	2742 (17.4)	2590 (16.4)	0.026
Sulfonylureas	1853 (10.1)	2152 (0.5)	0.437	1201 (7.6)	1039 (6.6)	0.04
DPP-4 inhibitors	1191 (6.5)	657 (0.2)	0.359	662 (4.2)	472 (3.0)	0.065
SGLT2 inhibitors	1017 (5.5)	327 (0.1)	0.335	443 (2.8)	268 (1.7)	0.075
Thiazolidinediones	338 (1.8)	311 (0.1)	0.182	205 (1.3)	181 (1.1)	0.014
Alpha glucosidase inhibitors	39 (0.2)	88 (0)	0.056	31 (0.2)	27 (0.2)	0.006
Estrogens	1998 (10.9)	57 627 (13.5)	0.082	1784 (11.3)	1767 (11.2)	0.003
Rivaroxaban	226 (1.2)	1626 (0.4)	0.095	192 (1.2)	204 (1.3)	0.007
Apixaban	266 (1.4)	1739 (0.4)	0.108	224 (1.4)	243 (1.5)	0.01
Warfarin	245 (1.3)	2491 (0.6)	0.077	198 (1.3)	228 (1.4)	0.016
Aspirin	1786 (9.7)	15 330 (3.6)	0.247	1417 (9.0)	1496 (9.5)	0.017
Clopidogrel	218 (1.2)	1146 (0.3)	0.108	182 (1.2)	205 (1.3)	0.013
Ticagrelor	52 (0.3)	158 (<0.1)	0.061	43 (0.3)	40 (0.3)	0.004
Prasugrel	24 (0.1)	47 (0)	0.045	17 (0.1)	18 (0.1)	0.002
Enoxaparin	1107 (6.0)	10 303 (2.4)	0.18	894 (5.7)	956 (6.1)	0.017
BMI						
Mean (SD)	41.0 (9.2)	30.4 (8.6)	1.189	40.7 (9.2)	40.1 (8.9)	0.062
<18.5	50 (0.3)	7204 (1.7)	0.144	45 (0.3)	43 (0.3)	0.002
18.5-<25.0	337 (1.8)	78 451 (18.4)	0.571	325 (2.1)	218 (1.4)	0.052
25.0-<30.0	1496 (8.1)	76 972 (18.1)	0.298	1354 (8.6)	1303 (8.3)	0.012
30.0-<40.0	6391 (34.7)	82 055 (19.2)	0.354	5373 (34.1)	5721 (36.3)	0.046
≥40	7213 (39.2)	36 026 (8.4)	0.773	5728 (36.4)	6187 (39.3)	0.06
HbA_1c_						
Mean (SD), %	7.5 (2.3)	5.7 (1.3)	0.958	7.2 (2.2)	6.5 (1.8)	0.314
<5.7	2914 (15.8)	35 435 (8.3)	0.232	2771 (17.6)	3459 (22.0)	0.11
5.7-<6.5	3221 (17.5)	13 692 (3.2)	0.482	2671 (17.0)	3133 (19.9)	0.076
≥6.5	6729 (36.5)	6881 (1.6)	0.992	4432 (28.1)	3853 (24.5)	0.084

Abbreviations: BMI, body mass index (calculated as weight in kilograms divided by height in meters squared); CKD, chronic kidney disease; DPP-4, dipeptidyl peptidase 4; ED, emergency department; GLP-1RA, glucagon-like peptide 1 receptor agonist; HbA_1c_, glycosylated hemoglobin; HIV, human immunodeficiency virus; PSM, propensity score matching; SERM, selective estrogen receptor modulators; SGLT2, sodium-glucose cotransporter 2; SMD, standardized mean difference; T2D, type 2 diabetes.

SI conversion factor: To convert hemoglobin A_1C_ to proportion of total hemoglobin, multiply by 0.01.

^a^ Covariates with patient counts fewer than 10 were not presented in this table, including dementia, SERM, dabigatran, and edoxaban, in accordance with the HIPAA Privacy Rule (Health Insurance Portability and Accountability Act).

^b^ SMD less than 0.10 indicates that the 2 comparison groups were well balanced.

^c^ Pacific Islander Includes Native Hawaiian or other Pacific Islander.

^d^ Separate listed option on the TriNetX platform.

^e^ Socioeconomic risks refer to persons with potential health hazards related to socioeconomic and psychosocial circumstances (*International Statistical Classification of Diseases and Related Health Problems, Tenth Revision *codes, Z55-Z65).

After applying PSM, there were 15 747 patients in each group (GLP-1RA plus progestins vs progestins alone). The mean (SD) age at index was 42.4 (10.0) years and 43.2 (12.0) years (SMD = 0.067). Race, ethnicity, comorbidities, BMI, and medication usage were comparable between the 2 groups (all SMDs <0.1). However, HbA_1c_ levels remained imbalanced after PSM. The GLP-1RA plus progestins group had a higher mean (SD) HbA_1c_ (7.2% [2.2%]) than the progestins only group (6.5% [1.8%]), with an SMD of 0.314, indicating a persistent difference in glycemic control despite similar T2D rates.

**Figure 2.**
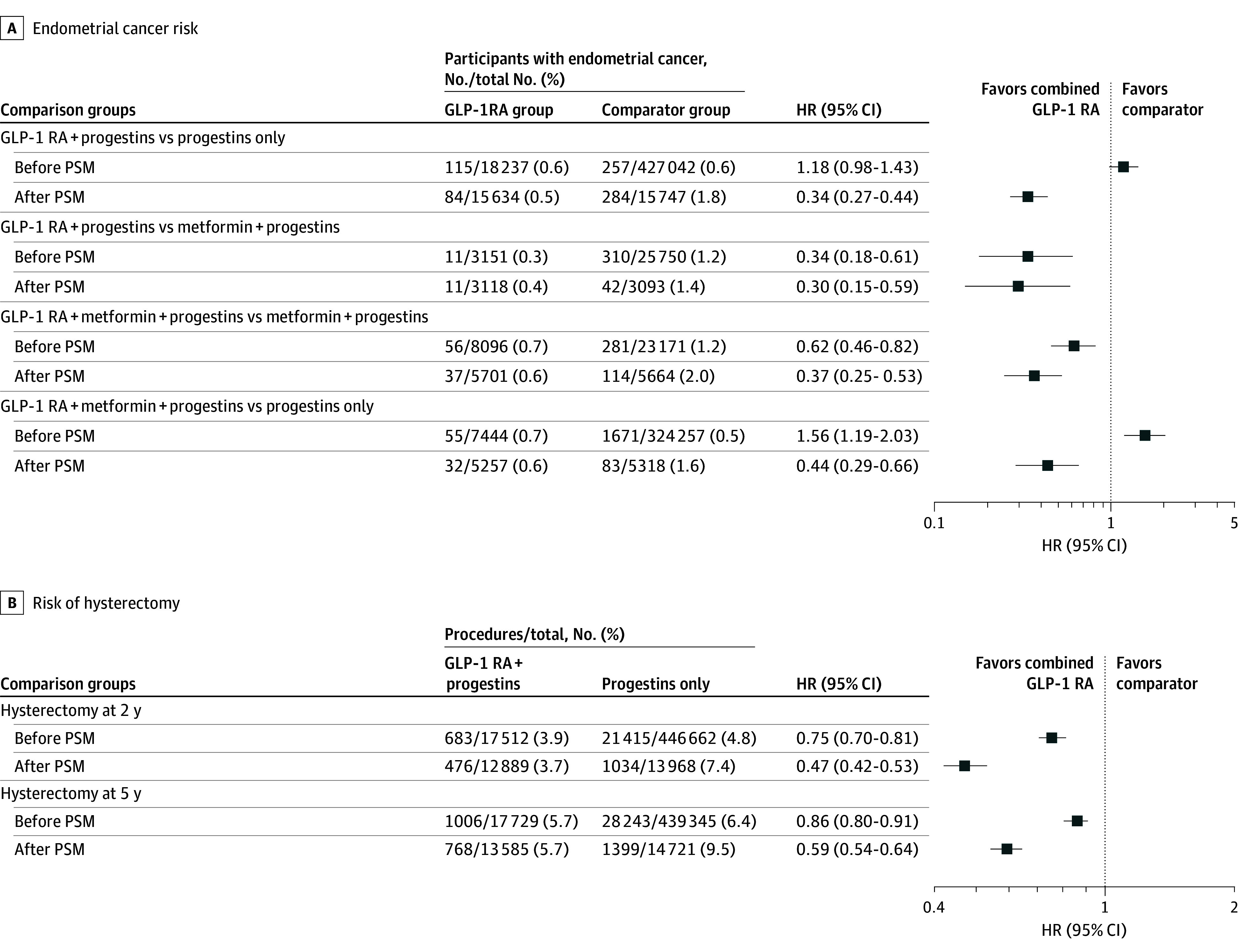
Hazard Ratios (HR) for Adding Glucagon-Like Peptide-1 Receptor Agonist (GLP-1RA) to Progestin Therapy and Endometrial Cancer Risk and Hysterectomy Outcomes All analyses exclude patients who had the outcome of interest prior to the observation window. In comparison A, 113 patients in the GLP-1RA plus progestins group were excluded from the HR analysis because their outcome occurred prior to the time-at-risk window. In comparison B, 19 patients in the GLP-1RA plus progestins group and 44 patients in the metformin plus progestins group were excluded for the same reason. In comparison C, 68 patients in the GLP-1RA, metformin, and progestins group and 105 patients in the metformin plus progestins group were excluded; and in comparison D, 61 patients in the GLP-1RA, metformin, and progestins group were excluded. PSM indicates propensity score matching.

The mean (SD) follow-up duration was 1332.5 (851.7) days and 1962.5 (1434.7) days, respectively (eTable 2 in Supplement 1). During follow-up, 84 of 15 634 patients (0.5%) in the GLP-1RA plus progestins group and 284 of 15 747 patients (1.8%) in the progestins only group developed EC (HR, 0.34 [95% CI: 0.27-0.44]), indicating a significantly lower risk of EC in the GLP-1RA plus progestins group (Figure 2A; eFigure, A in Supplement 1).

We repeated comparison A analysis using the US Collaborative Network. The effect sizes were similar, showing a lower risk of EC in the GLP-1RA plus progestins group compared with the progestins only group (eTable 3 in Supplement 1).

**Figure 3.**
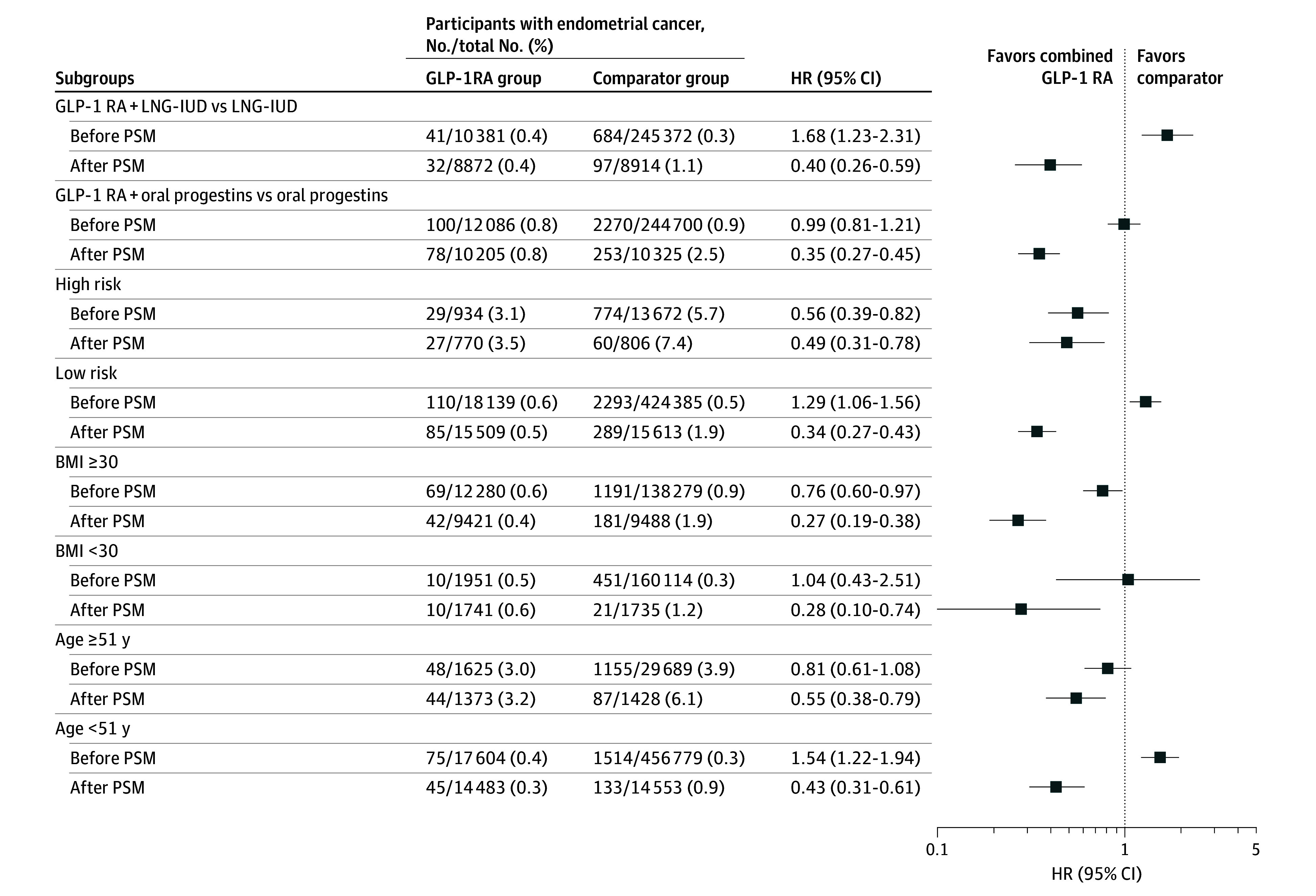
Hazard Ratios (HR) for Adding GLP-1RA to Progestin Therapy on Endometrial Cancer Risk (Subgroup Analyses of Comparison A) Subgroup analyses of endometrial cancer risk comparing glucagon-like peptide-1 receptor agonist (GLP-1RA) plus progestins vs progestins only, stratified by route of progestin administration, endometrial cancer risk level, body mass index (calculated as weight in kilograms divided by height in meters squared), and age. LNG-IUD indicates levonorgestrel-releasing intrauterine devices; PSM, propensity score matching.

We performed subgroup analysis in comparison A, stratifying patients by risk, the route of progestin administration, BMI, and age (Figure 3). In the subgroup of patients diagnosed with EH (ie, what we define as the high-risk group for this analysis), GLP-1RA plus progestins was associated with a lower risk of EC compared with progestins alone after PSM (HR, 0.49 [95% CI, 0.31-0.78]). A similar outcome was observed in the low-risk subgroup (defined as patients with benign uterine pathology), with a HR of 0.34 (95% CI, 0.27-0.43).

When examining the route of progestin administration, both local (LNG-IUD) and systemic (oral) progestins combined with GLP-1RA were associated with reduced EC risk compared with progestins alone. In the LNG-IUD subgroup, 32 of 8872 (0.4%) patients receiving GLP-1RA plus LNG-IUD developed EC, compared with 97 of 8914 (1.1%) in the LNG-IUD only group (HR, 0.40 [95% CI, 0.26-0.59]). In the systemic (oral) subgroup, 78 of 10 205 (0.8%) patients in the GLP-1RA plus oral progestin group developed EC, compared with 253 of 10 325 (2.5%) in the oral progestin only group (HR, 0.35 [95% CI, 0.27-0.45]).

Regardless of BMI stratification, the combination of GLP-1RA plus progestins was associated with a lower risk of EC. Among patients with a BMI of 30 or higher, EC occurred in 42 of 9421 patients (0.4%) in the GLP-1RA plus progestins group and 181 of 9488 (1.9%) in the progestins only group (HR, 0.27 [95% CI, 0.19-0.38]). Among individuals with a BMI under 30, 10 of 1741 (0.6%) in the GLP-1RA plus progestins group and 21 of 1735 (1.2%) in the progestins only group developed EC (HR, 0.28 [95% CI, 0.10-0.74]). The association remained unchanged when stratified by age 51 years (a proxy for menopause). Among patients aged 51 years or older, EC occurred in 44 of 1373 (3.2%) in the GLP-1RA plus progestins group vs 87 of 1428 (6.1%) in the progestins only group (HR, 0.55 [95% CI, 0.38-0.79]). In those under age 51, EC occurred in 45 of 14 483 (0.3%) in the GLP-1RA plus progestins group vs 133 of 14 553 (0.9%) in the progestins only group (HR, 0.43 [95% CI, 0.31-0.61]).

### Endometrial Cancer Risk in Patients Receiving GLP-1RA Plus Progestins Compared With Metformin Plus Progestins (Comparison B)

A total of 3165 patients who received GLP-1RA plus progestins and 25 957 patients who received metformin plus progestins were identified. The mean (SD) age at index was 42.3 (9.9) years in the GLP-1RA plus progestins group and 38.0 (11.5) years in the metformin plus progestins group. The baseline characteristics in comparison B are similar to those in comparison A (Table 2). After PSM, no significant imbalances remained among the measured covariates. The mean and median follow-up durations after PSM for EC diagnosis were similar to those in comparison A (eTable 2 in Supplement 1). There were 11 of 3118 patients (0.4%) in GLP-1RA plus progestins group and 42 of 3093 (1.4%) in metformin plus progestins group developed EC during the follow-up period (HR, 0.30 [95% CI, 0.15-0.59]), indicating a significantly lower risk in the GLP-1RA plus progestins group (Figure 2A; eFigure, B in Supplement 1).

**Table 2.** Patient Characteristics in Comparison B (GLP-1RA Plus Progestins vs Metformin Plus Progestins)

Characteristics^a^	Before PSM			After PSM		
	GLP-1RA + progestins, patients, No. (%) (n = 3165)	Metformin + progestins, patients, No. (%) (n = 25 957)	SMD^b^	GLP-1RA + progestins, patients, No. (%) (n = 3137)	Metformin + progestins, patients, No. (%) (n = 3137)	SMD^b^
Age at index, mean (SD), y	42.3 (9.9)	38.0 (11.5)	0.401	42.2 (9.8)	42.4 (12.4)	0.023
Race						
American Indian or Alaska Native	13 (0.4)	125 (0.5)	0.011	13 (0.4)	10 (0.3)	0.016
Asian	59 (1.9)	1566 (6.0)	0.215	59 (1.9)	66 (2.1)	0.016
Black or African American	949 (30.0)	6465 (24.9)	0.114	936 (29.8)	930 (29.6)	0.004
Pacific Islander^c^	15 (0.5)	295 (1.1)	0.074	15 (0.5)	25 (0.8)	0.04
White	1742 (55.0)	13 516 (52.1)	0.06	1728 (55.1)	1757 (56.0)	0.019
Other race^d^	97 (3.1)	1196 (4.6)	0.08	97 (3.1)	81 (2.6)	0.031
Unknown race	290 (9.2)	2794 (10.8)	0.053	289 (9.2)	273 (8.7)	0.018
Ethnicity						
Hispanic or Latino	360 (11.4)	4273 (16.5)	0.147	357 (11.4)	325 (10.4)	0.033
Not Hispanic or Latino	2389 (75.5)	17 866 (68.8)	0.149	2366 (75.4)	2394 (76.3)	0.021
Unknown ethnicity	416 (13.1)	3818 (14.7)	0.045	414 (13.2)	418 (13.3%)	0.004
Comorbidities						
Socioeconomic risks^e^	72 (2.3)	524 (2.0)	0.018	72 (2.3)	77 (2.5%)	0.01
Nicotine dependence	189 (6.0)	1783 (6.9)	0.037	189 (6.0)	183 (5.8%)	0.008
Hypertensive diseases	1169 (36.9)	6777 (26.1)	0.235	1142 (36.4)	1152 (36.7)	0.007
T2D	557 (17.6)	5958 (23.0)	0.133	543 (17.3)	545 (17.4)	0.002
T2D with complications	256 (8.1)	1837 (7.1)	0.038	244 (7.8)	226 (7.2)	0.022
Cerebrovascular diseases	42 (1.3)	361 (1.4)	0.006	42 (1.3)	31 (1.0)	0.033
Liver diseases	201 (6.4)	1233 (4.8)	0.07	197 (6.3)	189 (6.0)	0.011
Hyperlipidemia	530 (16.7)	2993 (11.5)	0.15	512 (16.3)	513 (16.4)	0.001
Acute myocardial infarction	21 (0.7)	108 (0.4)	0.034	21 (0.7)	18 (0.6)	0.012
Heart failure	123 (3.9)	468 (1.8)	0.126	115 (3.7)	121 (3.9)	0.01
Atherosclerosis	23 (0.7)	82 (0.3)	0.057	21 (0.7)	16 (0.5)	0.021
Peripheral vascular disease	27 (0.9)	105 (0.4)	0.057	25 (0.8)	27 (0.9)	0.007
CKD	183 (5.8)	400 (1.5)	0.227	169 (5.4)	172 (5.5)	0.004
Gastric ulcer	17 (0.5)	80 (0.3)	0.035	16 (0.5)	18 (0.6)	0.009
Connective tissue disorders	78 (2.5)	288 (1.1)	0.102	75 (2.4)	81 (2.6)	0.012
HIV disease	13 (0.4)	99 (0.4)	0.005	13 (0.4)	10 (0.3)	0.016
Chronic lower respiratory diseases	601 (19.0)	3456 (13.3)	0.155	597 (19.0)	592 (18.9)	0.004
Pregnancy	45 (1.4)	885 (3.4)	0.13	45 (1.4)	46 (1.5)	0.003
Service types, No. (%)						
Office or outpatient services	2363 (74.7)	15 588 (60.1)	0.315	2339 (74.6)	2324 (74.1)	0.011
Preventive medicine services	1052 (33.2)	5462 (21.0)	0.277	1040 (33.2)	1014 (32.3)	0.018
ED services	757 (23.9)	5556 (21.4)	0.06	745 (23.7)	740 (23.6)	0.004
Hospital inpatient services	191 (6.0)	1734 (6.7)	0.026	189 (6.0)	210 (6.7)	0.027
Medications, No. (%)						
Insulins	381 (12.0)	2298 (8.9)	0.104	369 (11.8)	386 (12.3)	0.017
Sulfonylureas	81 (2.6)	787 (3.0)	0.029	81 (2.6)	77 (2.5)	0.008
DPP-4 inhibitors	62 (2.0)	254 (1.0)	0.082	61 (1.9)	60 (1.9)	0.002
SGLT2 inhibitors	65 (2.1)	129 (0.5)	0.139	57 (1.8)	52 (1.7)	0.012
Thiazolidinediones	13 (0.4)	136 (0.5)	0.017	13 (0.4)	10 (0.3)	0.016
Estrogens	395 (12.5)	3471 (13.4)	0.027	395 (12.6)	364 (11.6)	0.03
Rivaroxaban	39 (1.2)	140 (0.5)	0.074	38 (1.2)	29 (0.9)	0.028
Apixaban	49 (1.5)	193 (0.7)	0.076	47 (1.5)	47 (1.5)	<0.001
Warfarin	42 (1.3)	273 (1.1)	0.025	41 (1.3)	36 (1.1)	0.014
Aspirin	213 (6.7)	1837 (7.1)	0.014	211 (6.7)	231 (7.4)	0.025
Clopidogrel	24 (0.8)	166 (0.6)	0.014	24 (0.8)	24 (0.8)	<0.001
Enoxaparin	169 (5.3)	1131 (4.4)	0.046	166 (5.3)	169 (5.4)	0.004
BMI						
Mean (SD)	40.0 (8.7)	37.9 (9.3)	0.237	40.0 (8.7)	39.7 (8.5)	0.038
18.5-<25	67 (2.1)	1423 (5.5)	0.177	67 (2.1)	60 (1.9)	0.016
25-<30	310 (9.8)	3178 (12.2)	0.078	309 (9.9)	302 (9.6)	0.008
30-<40	1256 (39.7)	8192 (31.6)	0.17	1244 (39.7)	1268 (40.4)	0.016
≥40	1172 (37.0)	6917 (26.6)	0.224	1155 (36.8)	1191 (38.0)	0.024
HbA_1c_, No. (%)						
Mean (SD), %	6.2 (1.8)	6.6 (1.8)	0.229	6.2 (1.8)	6.2 (1.7)	0.018
<5.7	834 (26.4)	3518 (13.6)	0.324	820 (26.1)	793 (25.3)	0.02
5.7-<6.5	459 (14.5)	4818 (18.6)	0.109	458 (14.6)	458 (14.6)	<0.001
≥6.5	421 (13.3)	4730 (18.2)	0.135	414 (13.2)	431 (13.7)	0.016

Abbreviations: BMI, body mass index (calculated as weight in kilograms divided by height in meters squared); CKD, chronic kidney disease; DPP-4, dipeptidyl peptidase 4; ED, emergency department; GLP-1RA, glucagon-like peptide 1 receptor agonist; HbA_1c_, glycosylated hemoglobin; HIV, human immunodeficiency virus; PSM, propensity score matching; SERM, selective estrogen receptor modulators; SGLT2, sodium-glucose cotransporter 2; SMD, standardized mean difference; T2D, type 2 diabetes.

SI conversion factor: To convert hemoglobin A_1C_ to proportion of total hemoglobin, multiply by 0.01.

^a^ Covariates with patient counts fewer than 10 were not presented in this table, including dementia, SERM, dabigatran, and edoxaban, in accordance with the HIPAA Privacy Rule (Health Insurance Portability and Accountability Act).

^b^ SMD less than 0.10 indicates that the 2 comparison groups were well balanced.

^c^ Pacific Islander Includes Native Hawaiian or other Pacific Islander.

^d^ Separate listed option on the TriNetX platform.

^e^ Socioeconomic risks refer to persons with potential health hazards related to socioeconomic and psychosocial circumstances (*International Statistical Classification of Diseases and Related Health Problems, Tenth Revision codes*, Z55-Z65).

### Endometrial Cancer Risk in Patients Receiving Triple Therapy (GLP-1RA, Metformin, and Progestins) Compared With Dual Therapy (Metformin Plus Progestins) (Comparison C) and Progestins Only (Comparison D)

After PSM, 5769 patients were included in each group of comparison C (GLP-1RA, metformin, and progestins vs metformin plus progestins), and 5318 in each group of comparison D (GLP-1RA, metformin, and progestins vs progestins only). The mean (SD) age at index was 43.3 (10.3) years in the GLP-1RA, metformin, and progestin group and 37.8 (11.6) years in the metformin plus progestin group. The mean (SD) age at index was 43.2 (10.3) years in the GLP-1RA plus progestin group and 35.1 (10.9) years in the progestin only group. The patient characteristics for comparisons C and D are shown in eTables 4 and 5 in Supplement 1, respectively. During the follow-up period in comparison C, EC developed in 37 of 5701 patients (0.6%) in the triple therapy group (GLP-1RA, metformin, and progestins) compared with 114 of 5664 patients (2.0%) in the dual therapy group (metformin plus progestin), with a HR of 0.37 (95% CI, 0.25-0.53). Similarly, in comparison D, 32 of 5257 patients (0.6%) in the triple therapy group and 83 of 5318 patients (1.6%) in monotherapy progestins only group developed EC (HR, 0.44 [95% CI, 0.29-0.66]) (Figure 2A; eFigure, C and D in Supplement 1).

### Subsequent Hysterectomy Outcomes

We evaluated the incidence of total hysterectomy as a secondary outcome in comparison A (GLP-1RA plus progestins vs progestins only), and found that the hysterectomy rate was significant lower in the GLP-1RA plus progestins group compared with the progestins only group, with similar HRs at 2-year and 5-year follow-ups (2-year follow-up: HR, 0.47 [95% CI, 0.42-0.53]; 5-year follow-up: HR, 0.59 [95% CI, 0.54-0.64]) (Figure 2B). We specifically evaluated the EIN subgroup (*ICD-10* code, N85.02) and found the consistent result (HR, 0.49 [95% CI, 0.24-1.00]).

## Discussion

In this large retrospective cohort study utilizing a clinical database, we found that the combination of GLP-1RA with progestin was associated with a significantly lower risk of developing EC compared with progestin alone in patients with benign uterine pathology or EH. This protective association remained consistent across subgroups stratified by BMI, age, baseline risk level, and progestin administration route. Specifically, both low risk (benign uterine pathology) and high risk (endometrial hyperplasia) demonstrated reduced EC incidence when treated with combined GLP-1RA and progestin compared with progestin alone. Furthermore, compared with metformin—another commonly used antidiabetic agent—the combination of GLP-1RA and progestin was associated with a lower risk of EC than combined metformin with progestin. Notably, patients receiving triple therapy (GLP-1RA, metformin, and progestin) exhibited a lower risk of EC development compared to those treated with either metformin and progestin or progestin only, suggesting that the addition of GLP-1RA may provide added or synergistic benefit in reducing EC risk. Lastly, we observed that patients treated with combined GLP-1RA and progestin had a lower rate of hysterectomy at 2-year and 5-year follow-up compared with those treated with progestin alone.

A prior study demonstrated that GLP-1RAs, combined with levonorgestrel, has been shown to significantly upregulate PR expression, reduce cell viability in EC organoids, and improve progestin responsiveness even in malignant neoplasms with low baseline PR levels.^17^ These data suggest that GLP-1RAs may help overcome progesterone resistance by inducing PR expression and activating downstream pathways such as PGRMC1, cyclic AMP, ERK, and c-Src.^17^ Our findings were consistent with these results. The combination of GLP-1RA with progestin was associated with a lower risk of EC development compared with progestins alone in both low- and high-risk patient subgroups. This risk reduction was observed regardless of the progestin administration route. In addition, our findings demonstrated that the combined use of GLP-1RA plus progestins significantly reduced EC risk not only in the endometrial hyperplasia subgroup (an association supported by previous preclinical data), but also in the low-risk population (benign uterine pathology). Additionally, subgroup analysis showed that the protective association between GLP-1RA with progestins and EC risk persisted across BMI categories and menopausal status (stratified at age 51 years). These findings suggest that GLP-1RAs may improve endometrial outcomes not only through metabolic regulation but also by potentially modulating hormonal signaling pathways.

Historical data demonstrated that metabolic dysfunction is associated with a higher risk of endometrial cancer.^21^ Notably, there were a greater proportion of patients with metabolic dysfunction among GLP-1RA users prior to PSM (Table 1). This may have resulted in a higher risk of endometrial cancer before PSM among GLP-1RA users in comparison A and comparison D. After adjusting these confounding factors, GLP-1RA was associated with a significantly lower risk of subsequent endometrial cancer. These findings were consistent with prior cohort studies,^22^ which also demonstrated that GLP-1RA is associated with lower risk of developing endometrial cancer.

Over the past decade, metformin has been studied as an adjunct to progestin for the treatment of EH and early-stage EC. A 2020 randomized clinical trial^23^ reported higher complete response rates and lower recurrence with metformin plus megestrol acetate compared with progestin alone. Similarly, a 2021 meta-analysis^24^ found reduced recurrence risk with combination therapy, though remission outcome was comparable. A smaller pilot study also showed a trend toward improved response with metformin, despite limited sample size.^25^ Collectively, these studies suggest that adding metformin to progestin therapy may improve histologic response and reduce recurrence risk. In our study, comparison B assessed EC risk between patients receiving GLP-1RA plus progestins vs metformin plus progestins. Results showed that combined GLP-1RA plus progestins was associated with a lower risk of EC development compared to combined metformin plus progestins. Furthermore, triple therapy of GLP-1RA, metformin, and progestin was associated with greater protective effect against EC development when compared with either combined metformin plus progestins or progestin alone, which further supports a potential synergetic role for GLP-1RA as an adjunct therapy in EC prevention among patients with EH or benign uterine pathology.

### Strengths and Limitations

The strengths of this study include the use of a large-scale, globally representative clinical dataset across multiple health care systems, and a comprehensive retrospective cohort design with PSM to minimize confounding by key baseline variables and to improve comparability between groups. Subgroup analyses provided nuanced insights into how GLP-1RA and progestin therapy may function across different patient populations. Additionally, the validation of results in an independent US-specific network reinforces the reproducibility of findings.

Several limitations should be considered. The retrospective nature of the analysis and reliance on diagnostic and procedural coding from EHR may introduce misclassification bias and an incomplete picture of clinical nuance. Histologic confirmation of disease regression or progression was not available. Unmeasured confounders such as medication adherence and durations, lifestyle behaviors, or socioeconomic status may have influenced the results. Lastly, because of the observational design, causal inference cannot be established. Future randomized clinical trials are needed to validate these associations prospectively.

## Conclusions

In this cohort study of women with benign uterine pathology or endometrial hyperplasia, adding GLP-1RA to progestin therapy was associated with lower endometrial cancer risk. Further prospective studies and clinical trials are warranted to validate these findings, to explore optimal dosing and duration strategies, and to better elucidate the biological mechanisms of GLP1-RA.

## Notes

Supplement 1. eMethods. eFigure. Cumulative incidence of endometrial cancer in four comparisons: Kaplan-Meier survival analysis eTable 1. Code descriptions for patient characteristics, inclusion, and exclusion criteria eTable 2. Mean, median, standard deviation, and interquartile range of follow-up time (in days) across four comparisons and hysterectomy outcomes. eTable 3. Patient characteristics in Comparison A (GLP-1RA + P versus P only) using data from the U.S Collaborative Network eTable 4. Patient characteristics in comparison C (GLP-1RA + metformin + P vs metformin + P) eTable 5. Patient characteristics in comparison D (GLP-1RA + metformin + P vs P only)

Supplement 2. Data Sharing Statement
