# Supplementary material for: GLP-1 Receptor Agonists Plus Progestins and Endometrial Cancer Risk in Nonmalignant Uterine Diseases
Source: JAMA Netw Open. 2026 Feb 10;9(2):e2558205. doi: 10.1001/jamanetworkopen.2025.58205 (PMC12892152; doi:10.1001/jamanetworkopen.2025.58205)
Supplement: Supplement 1. — eMethods. eFigure. Cumulative incidence of endometrial cancer in four comparisons: Kaplan-Meier survival analysis eTable 1. Code descriptions for patient characteristics, inclusion, and exclusion criteria eTable 2. Mean, median, standard deviation, and interquartile range of follow-up time (in days) across four comparisons and hysterectomy outcomes. eTable 3. Patient characteristics in Comparison A (GLP-1RA + P versus P only) using data from the U.S Collaborative Network eTable 4. Patient characteristics in comparison C (GLP-1RA + metformin + P vs metformin + P) eTable 5. Patient characteristics in comparison D (GLP-1RA + metformin + P vs P only) [file jamanetwopen-e2558205-s001.pdf]

Supplemental Online Content

Yen T, Hsieh TJ, Lee G, Toy EP, Wei JCC, Tanner EJ. GLP-1 Receptor agonists plus progestins on endometrial cancer risk in nonmalignant uterine diseases. *JAMA Netw Open.* 2026;9(2):e2558205. doi:10.1001/jamanetworkopen.2025.58205

eMethods.

eFigure 1. Cumulative incidence of endometrial cancer in four comparisons: Kaplan-Meier survival analysis

eTable 1. Code descriptions for patient characteristics, inclusion, and exclusion criteria

eTable 2. Mean, median, standard deviation, and interquartile range of follow-up time (in days) across four comparisons and hysterectomy outcomes.

eTable 3. Patient characteristics in Comparison A (GLP-1RA+P versus P only) using data from the U.S Collaborative Network

eTable 4. Patient characteristics in comparison C (GLP-1RA + metformin + P vs metformin + P)

eTable 5. Patient characteristics in comparison D (GLP-1RA + metformin + P vs P only)

This supplemental material has been provided by the authors to give readers additional information about their work.

© 2026 Yen T et al. *JAMA Network Open*.

eMethods.

Database

The TriNetX database includes patient demographics, clinical diagnoses recorded by the International Classification of Diseases, Tenth Revision (ICD-10) coding system, medication use, laboratory results, socioeconomic status, and more. This platform offers built-in analytic functions for comparisons. Comparisons were defined through query criteria via coding and analyzed within TriNetX platform.

Study design

Patient characteristics were extracted from TriNetX using diagnostic codes recorded within one year prior to the index event, including ICD-10, RxNorm, ATC, CPT, HCPCS, and SNOMED codes. Extracted variables included demographics (age, race, and ethnicity), comorbidities, body mass index (BMI), service types, pregnancy history, concomitant medication use, and laboratory results (Hemoglobin A1c). Race and ethnicity were assessed because racial and ethnic disparities in endometrial cancer are well documented, with Black women and other women of color facing higher incidence and lower survival. The corresponding codes are listed in eTable 1.

In the demographics category, patients were matched by age at index, race and ethnicity. In the diagnosis category, patients were matched by hypertensive diseases, type 2 diabetes mellitus (T2DM) with or without complications, cerebrovascular diseases, diseases of liver, nicotine dependence, hyperlipidemia, myocardial infarction, heart failure, diseases of arteries, atherosclerosis, peripheral vascular disease, dementia, chronic kidney disease, gastric ulcer, systemic connective tissue disorders, human immunodeficiency virus disease, chronic lower

© 2026 Yen T et al. *JAMA Network Open*.

respiratory diseases and pregnancy status. To account for patients' socioeconomic status (SES), we used the ICD-10 codes Z55 to Z65, "persons with potential health hazards related to socioeconomic and psychosocial circumstances", as a proxy measure for adverse socioeconomic determinants of health. In the procedure category, patients were matched by office or other outpatient services, preventive medicine services, emergency department services, and hospital inpatient and observation care services. In the medication category, patients were matched by metformin, insulin and analogues, sulfonylureas, dipeptidyl peptidase 4 (DPP-4) inhibitors, sodium-glucose co-transporter 2 (SGLT2) inhibitors, thiazolidinediones, alpha glucosidase inhibitors, estrogens, selective estrogen receptor modulators, dabigatran, rivaroxaban, apixaban, edoxaban, warfarin, aspirin, clopidogrel, ticagrelor, prasugrel, and enoxaparin characteristics. In the laboratory category, patients were matched by BMI (< 18.5, 18.5 to 24.9, 25 to 29.9, 30 to 39.9, and ≥ 40), and hemoglobin A1c (< 5.7%, 5.7% to 6.4%, and ≥ 6.5%).

© 2026 Yen T et al. *JAMA Network Open*.

eFigure. Cumulative incidence of endometrial cancer (EC) in four Comparisons: Kaplan–Meier survival analysis. **eFigure 1A.** Incidence of EC for patients receiving GLP-1RA+P versus P-only.

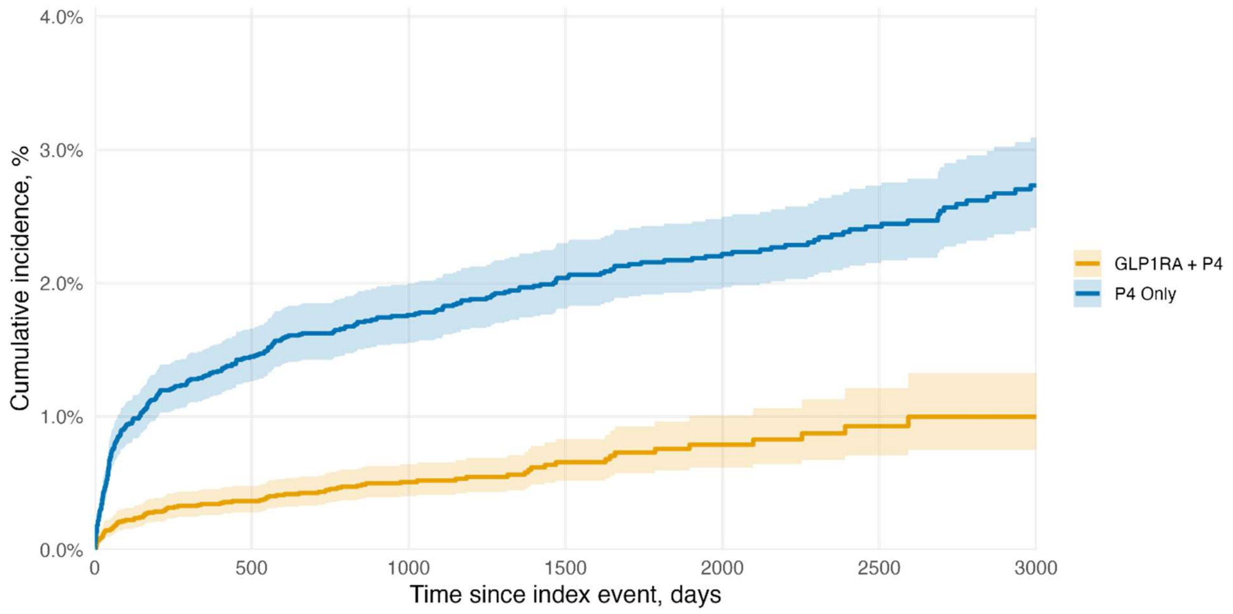

© 2026 Yen T et al. *JAMA Network Open*.

eFigure 1B. Incidence of EC for patients receiving GLP-1RA+P versus metformin+P.

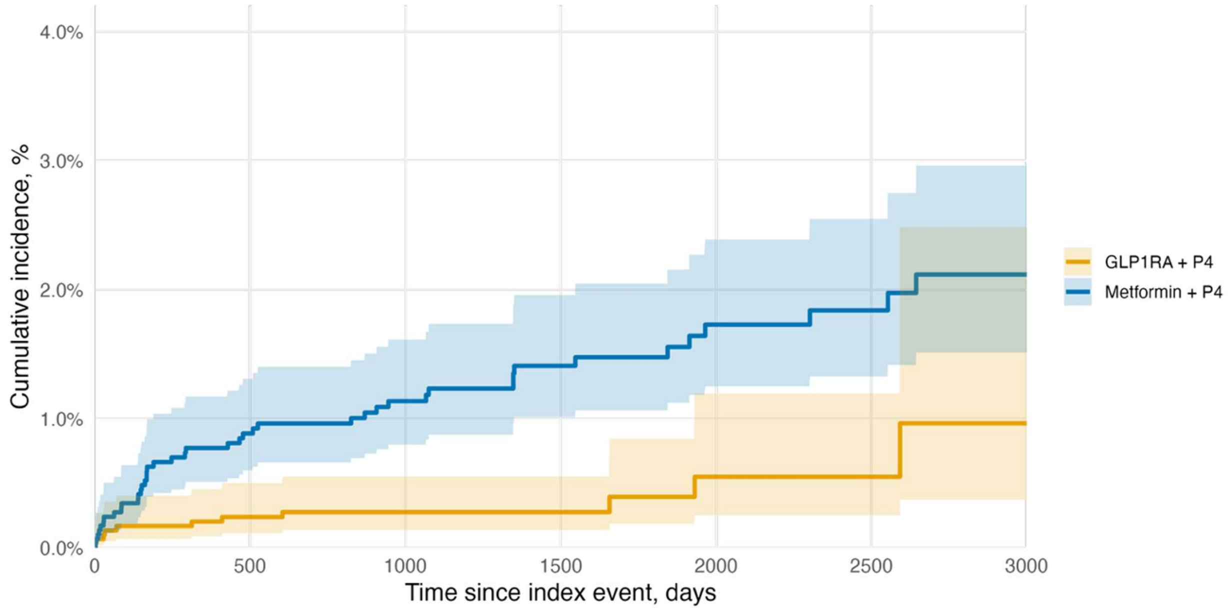

© 2026 Yen T et al. *JAMA Network Open*.

eFigure 1C. Incidence of EC for patients receiving GLP-1RA+metformin+P versus metformin+P.

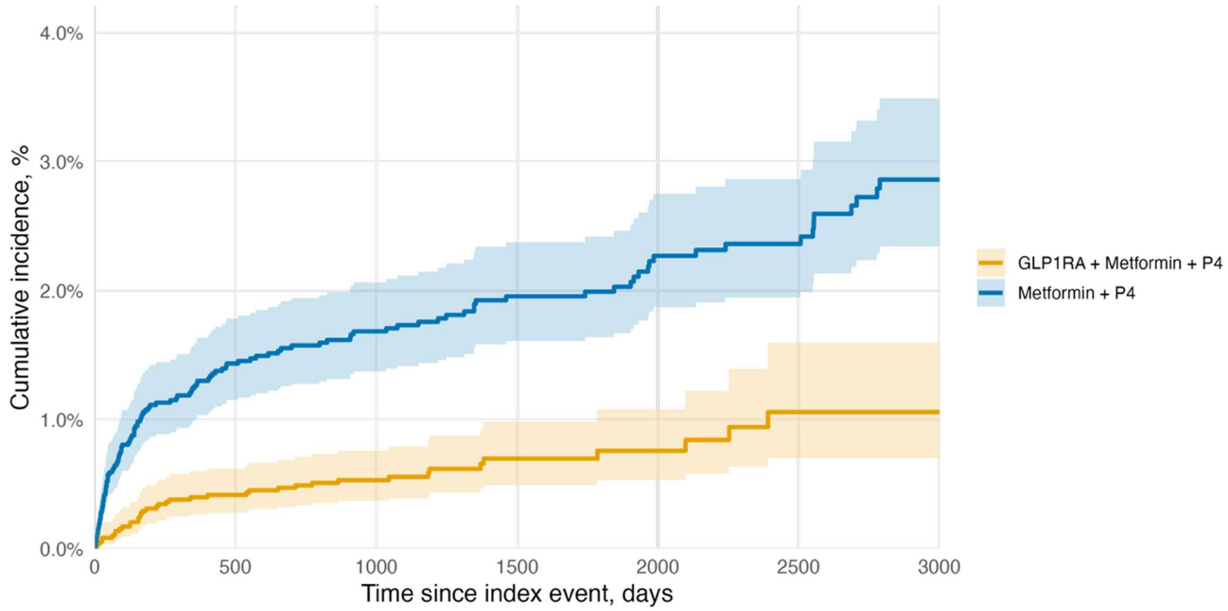

© 2026 Yen T et al. *JAMA Network Open*.

eFigure 1D. Incidence of EC for patients receiving GLP-1RA+metformin+P versus P-only.

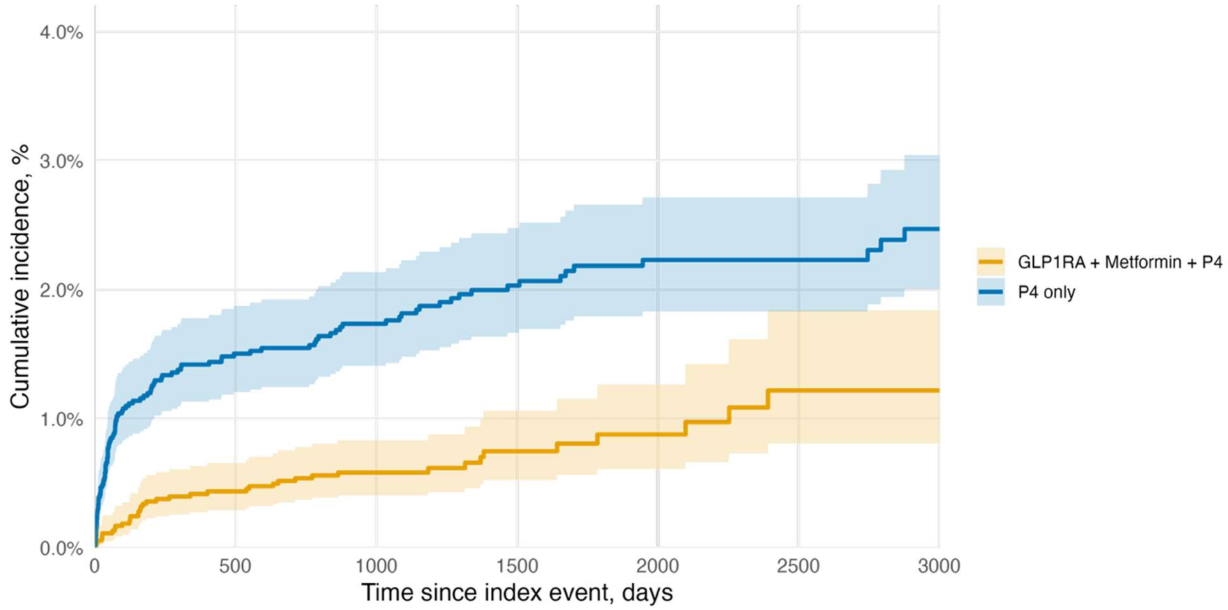

© 2026 Yen T et al. *JAMA Network Open*.

eTable 1. Code descriptions for patient characteristics, inclusion, and exclusion criteria

Variables	Code definitions
**Characteristics**	
Socioeconomic risks	ICD-10-CM: Z55-Z65
Nicotine dependence	ICD-10-CM: F17
Hypertensive diseases	ICD-10-CM: I10-I1A
T2DM	ICD-10-CM: E11, E11.6
Cerebrovascular diseases	ICD-10-CM: I60-I69
Liver diseases	ICD-10-CM: K70-K77
Hyperlipidemia	ICD-10-CM: E78.5
Acute MI	ICD-10-CM: I21
Heart failure	ICD-10-CM: I50
Atherosclerosis	ICD-10-CM: I70
Peripheral vascular disease	ICD-10-CM: I73.9
Dementia	ICD-10-CM: F03
CKD	ICD-10-CM: N18
Gastric ulcer	ICD-10-CM: K25
Connective tissue disorders	ICD-10-CM: M30-M36
HIV	ICD-10-CM: B20-B20
Chronic lower respiratory diseases	ICD-10-CM: J40-J4A
Pregnancy	ICD-10-PCS: 10
Office/ Outpatient Services	CPT: 1013626
Preventive Medicine Services	CPT: 1013829
ED Services	CPT: 1013711
Hospital Inpatient Services	CPT: 1013659
Insulins	ATC: A10A
Sulfonylureas	ATC: A10BB
DPP-4 inhibitors	ATC: A10BH
SGLT2 inhibitors	ATC: A10BK
Thiazolidinediones	ATC: A10BG
Alpha glucosidase inhibitors	ATC: A10BF
Estrogens	ATC: G03C
SERM	ATC: G03XC
Dabigatran	RxNorm: 1546356
Rivaroxaban	RxNorm: 1114195
Apixaban	RxNorm: 1364430
Edoxaban	RxNorm: 1599538
Warfarin	RxNorm: 11289
Aspirin	RxNorm: 1191

© 2026 Yen T et al. *JAMA Network Open*.

Clopidogrel	RxNorm: 32968
Ticagrelor	RxNorm: 1116632
Prasugrel	RxNorm: 613391
Enoxaparin	RxNorm: 67108
BMI	TriNetX curated code: 9083
HbA1c	TriNetX curated code: 9037
**Inclusion and exclusion**	
Endometrial hyperplasia	ICD-10-CM: N85.0, N85.00, N85.02
Benign uterine pathology	ICD-10-CM: N85.01(Simple endometrial hyperplasia); D25.0 (Submucosal myoma); N84.0 (Endometrial polyp); N92, N92.0, N92.1, N92.4, N92.5, N92.6, N93, N93.8, N93.9 (AUB)
GLP-1RA	ATC: A10BJ<br>RxNorm: 1991302 (Semaglutide), 475968 (Liraglutide), 1440051 (Lixisenatide), 1534763 (Albiglutide), 2601723 (Tirzepatide), 1551291 (Dulaglutide), 60548 (Exenatide)
Metformin	RxNorm: 6809
Megestrol acetate	RxNorm: 6703; HCPCS: S0179
Medroxyprogesterone	RxNorm: 6691
LNG-IUD	HCPCS: J7296, J7297, J7298, J7301, J7302, Q9984, S4981, S4989<br>CPT: 58300<br>ICD-10-CM: Z30.430, Z30.014, Z30.433, Z30.43, Z97.5, Z30.431<br>ICD-10-PCS: 0UH97HZ, 0UHC7HZ, 0UH98HZ, 0UHC8HZ<br>SNOMED: 65200003, 472837007, 176837007
Copper IUD [a]	HCPCS: J7300
Endometrial cancer	ICD-10-CM: C54.1, C54, C54.9, C54.3, C54.8, C54.0
Hysterectomy	CPT: 58150, 58152, 58200, 58210, 58260, 58262, 58263, 58267, 58270, 58275, 58280, 58285, 58290, 58291, 58292, 58293, 58294, 58548, 58550, 58552, 58553, 58554, 58570, 58571, 58572, 58573, 58575, 58953, 58954, 58956, 1013911, 1014205, 1014206, 1014210, 1014211, 1014212, 1014216, 1014640, 1018521, 1018522<br>ICD-10-PCS: 0UT90ZZ, 0UT9, 0UT94ZZ, 0UT97ZZ, 0UT98ZZ, 0UT9FZZ<br>SNOMED: 236886002, 35955002, 43791001, 54490004, 75835007, 86477000, 116140006, 116141005, 116142003, 116143008, 116144002, 236887006, 236888001, 236891001, 265056007, 302190000, 302191001, 307771009, 359977003, 413144006, 413145007, 414575003, 441820006, 446446002, 447771005, 448539002, 699789005, 708877008, 708878003, 708985003, 739674007, 762625001, 767610009, 767612001, 1303414001

Abbreviations: AUB, abnormal uterine bleeding; BMI, body mass index; CKD, chronic kidney disease; DPP-4, Dipeptidyl peptidase 4; ED, emergency department; GLP1-RA, Glucagon-like peptide-1 receptor agonist; HbA1c, Hemoglobin A1c; HIV, Human immunodeficiency virus disease; ICD-10, the International Classification of Diseases, Tent Revision. MI, myocardial

© 2026 Yen T et al. *JAMA Network Open*.

infarction; SERM, selective estrogen receptor modulator; SGLT2, Sodium-glucose co-transporter 2; T2DM, Type 2 diabetes mellitus; LNG-IUD, Levonorgestrel-releasing intrauterine device. Socioeconomic risks refer to persons with potential health hazards related to socioeconomic and psychosocial circumstances. [a], Copper intrauterine devices were excluded from the query of IUD users in this study.

© 2026 Yen T et al. *JAMA Network Open*.

eTable 2. Mean, median, standard deviation, and interquartile range of follow-up time (in days) across four comparisons and hysterectomy outcomes.

		Before PSM				After PSM			
		Mean Follow-up (Days)	SD	Median Follow-up (Days)	IQR	Mean Follow-up (Days)	SD	Median Follow-up (Days)	IQR
**Comparison A**	GLP-1RA+P	1372.184	868.549	1110	900	1332.547	851.685	1070	847
	P-only	1964.491	1519.692	1634	1976	1962.505	1434.73	1648	1843
**Comparison B**	GLP-1RA+P	1307.244	782.482	1099	863	1307.737	783.616	1099	863
	Metformin+P	1818.81	1365.899	1488	1721	1823.176	1297.767	1525	1661
**Comparison C**	GLP-1RA+metformin+P	1491.835	910.447	1237	980	1438.061	901.342	1183	903
	Metformin+P	1850.718	1375.456	1525	1738	1952.777	1413.113	1637	1839
**Comparison D**	GLP-1RA+metformin+P	1491.478	907.972	1236	980	1405.905	873.515	1154.5	868
	P-only	1952.78	1506.169	1633	1935	1868.296	1376.169	1572	1713
**Hyst at 2 years**	GLP-1RA+P	694.792	124.511	730	0	691.579	129.154	730	0
	P-only	635.955	215.013	730	0	665.927	174.400	730	0
**Hyst at 5 years**	GLP-1RA+P	1270.737	458.213	1237	866	1239.436	460.659	1187	810
	P-only	1328.761	620.055	1714	911	1370.265	560.664	1663	824

Abbreviations: Hyst, hysterectomy; IQR, interquartile range; GLP-1RA, glucagon-like peptide-1 receptor agonist; P, progestins; PSM, propensity score matching; SD, standard deviation.

© 2026 Yen T et al. *JAMA Network Open*.

eTable 3. Risk of endometrial cancer and patient characteristics in Comparison A (GLP-1RA+P versus P-only) using data from the U.S. Collaborative Network.

	Before PSM			After PSM		
**GLP-1RA+P versus P-only**	**GLP-1RA+P EC/ Total, n**	**P-only EC/ Total, n**	**HR (95% CI)**	**GLP-1RA+P EC/ Total, n**	**P-only EC/ Total, n**	**HR (95% CI)**
	114/ 18,103	2,319/ 397,891	1.23 (1.02, 1.49)	87/ 15,393	297/ 15,504	0.34 (0.26, 0.43)
**Characteristics**	**GLP-1RA+P (n = 18,221)**	**P-only (n = 397,891)**	**SMD [a]**	**GLP-1RA+P (n = 15,504)**	**P-only (n = 15,504)**	**SMD [a]**
Age at Index, mean (SD)	43.1 (10.1)	34.9 (10.9)	0.777	42.4 (10.0)	43.1 (11.9)	0.067
Race, No. (%)						
Native American	109 (0.60%)	1,621 (0.40%)	0.027	95 (0.60%)	56 (0.40%)	0.036
Asian	447 (2.50%)	17,291 (4.30%)	0.105	381 (2.50%)	350 (2.30%)	0.013
Black or African American	5,424 (29.80%)	88,283 (22.20%)	0.174	4,543 (29.30%)	4,577 (29.50%)	0.005
Pacific Islander	182 (1.00%)	4,118 (1.00%)	0.004	148 (1.00%)	173 (1.10%)	0.016
White	10,062 (55.20%)	234,351 (58.90%)	0.074	8,599 (55.50%)	8,627 (55.60%)	0.004
Other Race	678 (3.70%)	17,914 (4.50%)	0.039	590 (3.80%)	607 (3.90%)	0.006
Unknown Race	1,319 (7.20%)	34,313 (8.60%)	0.051	1,148 (7.40%)	1,114 (7.20%)	0.008
Ethnicity, No. (%)						
Hispanic or Latino	2,265 (12.40%)	54,834 (13.80%)	0.04	1,970 (12.70%)	1,976 (12.70%)	0.001
Not Hispanic or Latino	13,814 (75.80%)	283,707 (71.30%)	0.102	11,654 (75.20%)	11,634 (75.00%)	0.003
Unknown Ethnicity	2,142 (11.80%)	59,350 (14.90%)	0.093	1,880 (12.10%)	1,894 (12.20%)	0.003
Comorbidities, No. (%)						
Socioeconomic risks	503 (2.80%)	8,964 (2.30%)	0.032	392 (2.50%)	387 (2.50%)	0.002
Nicotine dependence	1,367 (7.50%)	23,138 (5.80%)	0.068	1,104 (7.10%)	1,111 (7.20%)	0.002
Hypertensive diseases	8,566 (47.00%)	41,815 (10.50%)	0.881	6,533 (42.10%)	6,721 (43.40%)	0.025
T2DM	8,538 (46.90%)	13,341 (3.40%)	1.16	5,933 (38.30%)	5,689 (36.70%)	0.033
T2DM with complications	4,313 (23.70%)	3,906 (1.00%)	0.735	2,635 (17.00%)	2,271 (14.60%)	0.064
Cerebrovascular diseases	350 (1.90%)	3,131 (0.80%)	0.098	294 (1.90%)	326 (2.10%)	0.015
Liver diseases	1,693 (9.30%)	6,781 (1.70%)	0.338	1,234 (8.00%)	1,209 (7.80%)	0.006
Hyperlipidemia	4,415 (24.20%)	15,306 (3.80%)	0.614	3,177 (20.50%)	3,127 (20.20%)	0.008

© 2026 Yen T et al. *JAMA Network Open*.

Acute MI	164 (0.90%)	897 (0.20%)	0.09	127 (0.80%)	149 (1.00%)	0.015
Heart failure	709 (3.90%)	3,098 (0.80%)	0.207	570 (3.70%)	590 (3.80%)	0.007
Atherosclerosis	143 (0.80%)	666 (0.20%)	0.09	121 (0.80%)	136 (0.90%)	0.011
Peripheral vascular disease	171 (0.90%)	683 (0.20%)	0.103	128 (0.80%)	149 (1.00%)	0.014
CKD	855 (4.70%)	4,238 (1.10%)	0.218	663 (4.30%)	720 (4.60%	0.018
Gastric ulcer	84 (0.50%)	711 (0.20%)	0.05	71 (0.50%)	70 (0.50%)	0.001
Connective tissue disorders	396 (2.20%)	4,078 (1.00%)	0.092	318 (2.10%)	323 (2.10%)	0.002
HIV disease	103 (0.60%)	1,240 (0.30%)	0.038	79 (0.50%)	79 (0.50%)	<0.001
Chronic lower respiratory diseases	3,775 (20.70%)	34,475 (8.70%)	0.346	2,966 (19.10%)	3,065 (19.80%)	0.016
Pregnancy	241 (1.3%)	31,067 (7.80%)	0.315	232 (1.50%)	225 (1.50%)	0.004
Service types, No. (%)						
Office/ Outpatient Services	13,589 (74.60%)	207,450 (52.10%)	0.479	11,261 (72.60%)	11,306 (72.90%)	0.007
Preventive Medicine Services	5,578 (30.60%)	82,290 (20.70%)	0.229	4,645 (30.00%)	4,569 (29.50%)	0.011
ED Services	4,601 (25.30%)	74,231 (18.70%)	0.16	3,741 (24.10%)	3,737 (24.10%)	0.001
Hospital Inpatient Services	1,288 (7.10%)	22,631 (5.70%)	0.057	1,041 (6.70%)	1,063 (6.90%)	0.006
Medications, No. (%)						
Metformin	7,150 (39.20%)	9,771 (2.50%)	1.016	4,866 (31.40%)	4,633 (29.90%)	0.033
Insulins	3,942 (21.60%)	8,354 (2.10%)	0.634	2,702 (17.40%)	2,557 (16.50%)	0.025
Sulfonylureas	1,825 (10.00%)	2,064 (0.50%)	0.435	1,156 (7.50%)	990 (6.40%)	0.042
DPP-4 inhibitors	1,149 (6.30%)	568 (0.10%)	0.354	609 (3.90%)	419 (2.70%)	0.068
SGLT2 inhibitors	1,007 (5.50%)	295 (0.10%)	0.335	425 (2.70%)	246 (1.60%)	0.079
Thiazolidinediones	324 (1.80%)	284 (0.10%)	0.179	198 (1.30%)	185 (1.20%)	0.008
Alpha glucosidase inhibitors	30 (0.20%)	61 (0.00%)	0.05	21 (0.10%)	16 (0.10%)	0.009
Estrogens	1,966 (10.80%)	56,314 (14.20%)	0.102	1,729 (11.20%)	1,684 (10.90%)	0.009
Rivaroxaban	224 (1.20%)	1,607 (0.40%)	0.092	185 (1.20%)	216 (1.40%)	0.018
Apixaban	265 (1.50%)	1,719 (0.40%)	0.106	220 (1.40%)	231 (1.50%)	0.006
Warfarin	242 (1.30%)	2,454 (0.60%)	0.073	202 (1.30%)	233 (1.50%)	0.017
Aspirin	1,774 (9.70%)	14,928 (3.80%)	0.24	1,384 (8.90%)	1,433 (9.20%)	0.011
Clopidogrel	208 (1.10%)	1,096 (0.30%)	0.103	173 (1.10%)	199 (1.30%)	0.015
Ticagrelor	52 (0.30%)	153 (0.00%)	0.061	38 (0.20%)	35 (0.20%)	0.004
Prasugrel	23 (0.10%)	46 (0.00%)	0.044	15 (0.10%)	16 (0.10%)	0.002
Enoxaparin	1,112 (6.10%)	10,202 (2.60%)	0.174	896 (5.80%)	945 (6.10%)	0.013
BMI, mean (SD), kg/m^2	41.1 (9.2)	30.5 (8.6)	1.187	40.7 (9.2)	40.3 (8.9)	0.042

© 2026 Yen T et al. *JAMA Network Open*.

< 18.50, No. (%)	50 (0.30%)	6,944 (1.7%)	0.148	43 (0.30%)	39 (0.30%)	0.005
18.50 – 25, No. (%)	320 (1.80%)	76,842 (19.3%)	0.597	309 (2.00%)	191 (1.20%)	0.06
25 – 30, No. (%)	1,475 (8.10%)	76,323 (19.2%)	0.327	1,345 (8.70%)	1,202 (7.80%)	0.034
30 – 40, No. (%)	6,368 (34.90%)	81,706 (20.5%)	0.326	5,337 (34.40%)	5,606 (36.20%)	0.036
≥ 40, No. (%)	7,205 (39.50%)	35,963 (9.0%)	0.761	5,648 (36.40%)	6,211 (40.10%)	0.075
HbA1c, mean (SD), %	7.5 (2.3)	5.7 (1.3)	0.958	7.1 (2.2)	6.6 (1.8)	0.286
< 5.70, No. (%)	2,900 (15.90%)	34,993 (8.80%)	0.218	2,775 (17.90%)	3,362 (21.70%)	0.095
5.70 - 6.50, No. (%)	3,196 (17.50%)	13,376 (3.40%)	0.476	2,655 (17.10%)	3,078 (19.90%)	0.07
≥ 6.50, No. (%)	6,677 (36.60%)	6,705 (1.70%)	0.991	4,343 (28.00%)	3,787 (24.40%)	0.082

Abbreviations: BMI, body mass index; CKD, Chronic kidney disease; CI, confidence interval; DPP-4, Dipeptidyl peptidase 4; EC, endometrial cancer; ED, emergency department; GLP-1RA, glucagon-like peptide-1 receptor agonist; HbA1c, glycosylated hemoglobin; HIV, Human immunodeficiency virus; HR, hazard ratio; MI, myocardial infarction; P, progestins; PSM, propensity score matching; SD, standard deviation; SERM, Selective estrogen receptor modulators; SGLT2, Sodium-glucose co-transporter 2; SMD, standardized mean difference; T2DM, Type 2 diabetes mellitus. [a], SMD less than 0.10 indicates that the two comparison groups were well balanced. Native American refers to American Indian or Alaska Native; Pacific Islander refers to Native Hawaiian or Other Pacific Islander. In accordance with the HIPAA Privacy Rule, covariates with fewer than 10 patients were not presented in this table, including dementia and the use of SERM, dabigatran, and edoxaban.

© 2026 Yen T et al. *JAMA Network Open*.

eTable 4. Patient characteristics in Comparison C (GLP-1RA+metformin+P versus Metformin+P).

Characteristics	Before PSM			After PSM		
	GLP-1RA+metformin+P (n = 8,199)	Metformin+P (n = 23,337)	SMD [a]	GLP-1RA+metformin+P (n = 5,769)	Metformin+P (n = 5,769)	SMD [a]
Age at Index, mean (SD)	43.3 (10.3)	37.8 (11.6)	0.498	41.9 (10.2)	42.3 (12.1)	0.042
Race, No. (%)						
Native American	57 (0.7%)	116 (0.5%)	0.026	46 (0.8%)	32 (0.6%)	0.03
Asian	196 (2.4%)	1,318 (5.6%)	0.166	154 (2.7%)	139 (2.4%)	0.017
Black or African American	2,427 (29.6%)	5,697 (24.4%)	0.117	1,623 (28.1%)	1,677 (29.1%)	0.021
Pacific Islander	42 (0.5%)	149 (0.6%)	0.017	23 (0.4%)	46 (0.8%)	0.052
White	4,487 (54.7%)	12,302 (52.7%)	0.04	3,157 (54.7%)	3,194 (55.4%)	0.013
Other Race	372 (4.5%)	1,245 (5.3%)	0.037	275 (4.8%)	227 (3.9%)	0.041
Unknown Race	618 (7.5%)	2,510 (10.8%)	0.112	491 (8.5%)	454 (7.9%)	0.023
Ethnicity, No. (%)						
Hispanic or Latino	1,020 (12.4%)	3,501 (15%)	0.074	765 (13.3%)	774 (13.4%)	0.005
Not Hispanic or Latino	6,221 (75.9%)	16,448 (70.5%)	0.122	4,277 (74.1%)	4,298 (74.5%)	0.008
Unknown Ethnicity	958 (11.7%)	3,388 (14.5%)	0.084	727 (12.6%)	697 (12.1%)	0.016
Comorbidities, No. (%)						
Socioeconomic risks	244 (3%)	466 (2%)	0.063	139 (2.4%)	143 (2.5%)	0.004
Nicotine dependence	737 (9%)	1,697 (7.3%)	0.063	473 (8.2%)	462 (8%)	0.007
Hypertensive diseases	4,184 (51%)	5,998 (25.7%)	0.539	2,466 (42.7%)	2,528 (43.8%)	0.022
T2DM	4,455 (54.3%)	4,567 (19.6%)	0.772	2,407 (41.7%)	2,453 (42.5%)	0.016
T2DM with complications	2,481 (30.3%)	1,529 (6.6%)	0.643	1,066 (18.5%)	1,083 (18.8%)	0.008
Cerebrovascular diseases	160 (2.0%)	323 (1.4%)	0.044	112 (1.9%)	109 (1.9%)	0.004
Liver diseases	875 (10.7%)	1,071 (4.6%)	0.231	466 (8.1%)	470 (8.1%)	0.003
Hyperlipidemia	2,239 (27.3%)	2,700 (11.6%)	0.406	1,238 (21.5%)	1,228 (21.3%)	0.004
Acute MI	68 (0.8%)	99 (0.4%)	0.051	42 (0.7%)	47 (0.8%)	0.01
Heart failure	299 (3.6%)	403 (1.7%)	0.119	184 (3.2%)	194 (3.4%)	0.01
Atherosclerosis	57 (0.7%)	75 (0.3%)	0.053	35 (0.6%)	36 (0.6%)	0.002
Peripheral vascular disease	79 (1%)	95 (0.4%)	0.067	46 (0.8%)	46 (0.8%)	<0.001
CKD	340 (4.1%)	353 (1.5%)	0.159	178 (3.1%)	186 (3.2%)	0.008
Gastric ulcer	32 (0.4%)	71 (0.3%)	0.015	24 (0.4%)	20 (0.3%)	0.011
Connective tissue disorders	163 (2%)	257 (1.1%)	0.072	110 (1.9%)	101 (1.8%)	0.012
HIV disease	41 (0.5%)	86 (0.4%)	0.02	23 (0.4%)	22 (0.4%)	0.003

© 2026 Yen T et al. *JAMA Network Open*.

Chronic lower respiratory diseases	1,847 (22.5%)	3,195 (13.7%)	0.231	1,127 (19.5%)	1,155 (20%)	0.012
Pregnancy	116 (1.4%)	830 (3.6%)	0.138	102 (1.8%)	102 (1.8%)	<0.001
Service types, No. (%)						
Office/ Outpatient Services	6,242 (76.1%)	14,360 (61.5%)	0.319	4,178 (72.4%)	4,103 (71.1%)	0.029
Preventive Medicine Services	2,425 (29.6%)	5,136 (22%)	0.174	1,589 (27.5%)	1,523 (26.4%)	0.026
ED Services	2,177 (26.6%)	4,821 (20.7%)	0.139	1,402 (24.3%)	1,386 (24%)	0.006
Hospital Inpatient Services	617 (7.5%)	1,557 (6.7%)	0.033	423 (7.3%)	439 (7.6%)	0.011
Medications, No. (%)						
Metformin	4,740 (57.8%)	4,924 (21.1%)	0.81	2,609 (45.20%)	2,525 (43.80%)	0.029
Insulins	2,036 (24.8%)	2,014 (8.6%)	0.445	1,031 (17.9%)	1,082 (18.8%)	0.023
Sulfonylureas	1,070 (13.1%)	675 (2.9%)	0.382	459 (8%)	461 (8%)	0.001
DPP-4 inhibitors	621 (7.6%)	225 (1%)	0.331	198 (3.4%)	185 (3.2%)	0.013
SGLT2 inhibitors	589 (7.2%)	115 (0.5%)	0.354	128 (2.2%)	110 (1.9%)	0.022
Thiazolidinediones	164 (2%)	114 (0.5%)	0.137	76 (1.30%)	77 (1.30%)	0.002
Alpha glucosidase inhibitors	22 (0.3%)	24 (0.1%)	0.038	10 (0.20%)	10 (0.20%)	<0.001
Estrogens	805 (9.8%)	3,108 (13.3%)	0.11	640 (11.10%)	588 (10.20%)	0.029
Rivaroxaban	86 (1%)	125 (0.5%)	0.058	52 (0.90%)	61 (1.10%)	0.016
Apixaban	112 (1.4%)	176 (0.8%)	0.06	76 (1.30%)	75 (1.30%)	0.002
Warfarin	110 (1.3%)	249 (1.1%)	0.025	70 (1.20%)	85 (1.50%)	0.023
Aspirin	851 (10.4%)	1,675 (7.2%)	0.113	529 (9.20%)	551 (9.60%)	0.013
Clopidogrel	99 (1.2%)	148 (0.6%)	0.06	72 (1.20%)	74 (1.30%)	0.003
Ticagrelor	23 (0.3%)	24 (0.1%)	0.041	13 (0.20%)	14 (0.20%)	0.004
Enoxaparin	539 (6.6%)	1,026 (4.4%)	0.096	360 (6.20%)	379 (6.60%)	0.013
BMI, mean (SD), kg/m^2	41.8 (9.4)	38.1 (9.3)	0.394	41.5 (9.5)	40.8 (9.1)	0.076
< 18.5, No. (%)	66 (0.8%)	198 (0.8%)	0.005	45 (0.80%)	47 (0.80%)	0.004
18.5 – 25, No. (%)	123 (1.5%)	1,286 (5.5%)	0.219	107 (1.90%)	84 (1.50%)	0.031
25 – 30, No. (%)	611 (7.5%)	2,863 (12.3%)	0.162	476 (8.30%)	446 (7.70%)	0.019
30 – 40, No. (%)	2,771 (33.8%)	7,568 (32.4%)	0.029	1,921 (33.30%)	1,842 (31.90%)	0.029
≥ 40, No. (%)	3,431 (41.8%)	6,539 (28%)	0.293	2,222 (38.50%)	2,212 (38.30%)	0.004
HbA1c, mean (SD), %	7.8 (2.3)	6.6 (1.7)	0.589	7.4 (2.2)	7.0 (1.9)	0.167
< 5.7, No. (%)	1,063 (13%)	3,361 (14.4%)	0.042	909 (15.80%)	887 (15.40%)	0.011
5.7 - 6.5, No. (%)	1,735 (21.2%	4,560 (19.5%	0.04	1,306 (22.60%)	1,305 (22.60%)	<0.001
≥ 6.5, No. (%)	4,106 (50.1%)	4,446 (19.1%)	0.69	2,133 (37.00%)	2,136 (37.00%)	0.001

© 2026 Yen T et al. *JAMA Network Open*.

Abbreviations: BMI, body mass index; CKD, Chronic kidney disease; DPP-4, Dipeptidyl peptidase 4; ED, emergency department; GLP-1RA, glucagon-like peptide 1 receptor agonist; HbA1c, glycosylated hemoglobin; HIV, Human immunodeficiency virus; MI, myocardial infarction; P, progestins; PSM, propensity score matching; SD, standard deviation; SERM, selective estrogen receptor modulators; SGLT2, Sodium-glucose co-transporter 2; SMD, standardized mean difference; T2DM, Type 2 diabetes mellitus. [a], SMD less than 0.10 indicates that the two comparison groups were well balanced. Native American refers to American Indian or Alaska Native; Pacific Islander refers to Native Hawaiian or Other Pacific Islander. In accordance with the HIPAA Privacy Rule, covariates with fewer than 10 patients were not presented in this table, including dementia and the use of SERM, dabigatran, edoxaban, and prasugrel.

© 2026 Yen T et al. *JAMA Network Open*.

eTable 5. Patient characteristics in Comparison D (GLP-1RA+metformin+P versus P-only).

	Before PSM			After PSM		
**Characteristics**	**GLP-1RA+Metformin+P (n = 8,376)**	**P-only (n = 362,574)**	**SMD [a]**	**GLP-1RA+Metformin+P (n = 5,318)**	**P-only (n = 5,318)**	**SMD [a]**
Age at Index, mean (SD)	43.2 (10.3)	35.1 (10.9)	0.766	41.8 (10.3)	42.6 (12.2)	0.07
Race, No. (%)						
Native American	60 (0.7%)	1,354 (0.4%)	0.047	41 (0.8%)	24 (0.5%)	0.041
Asian	196 (2.3%)	20,113 (5.5%)	0.165	128 (2.4%)	95 (1.8%)	0.043
Black or African American	2,519 (30.1%)	74,344 (20.5%)	0.222	1,536 (28.9%)	1,564 (29.4%)	0.012
Pacific Islander	42 (0.5%)	1,643 (0.5%)	0.007	21 (0.4%)	46 (0.9%)	0.059
White	4,567 (54.5%)	204,310 (56.3%)	0.037	2,919 (54.9%)	3,022 (54.9%)	0.039
Other Race	378 (4.5%)	16,892 (4.7%)	0.007	244 (4.6%)	225 (4.2%)	0.017
Unknown Race	614 (7.3%)	43,918 (12.1%)	0.162	429 (8.1%)	342 (6.4%)	0.063
Ethnicity, No. (%)						
Hispanic or Latino	1,029 (12.3%)	40,901 (11.3%)	0.031	643 (12.1%)	674 (12.7%)	0.018
Not Hispanic or Latino	6,389 (76.3%)	253,131 (69.8%)	0.146	3,994 (75.1%)	4,023 (75.6%)	0.013
Unknown Ethnicity	958 (11.4%)	68,542 (18.9%)	0.209	681 (12.8%)	621 (11.7%)	0.034
Comorbidities, No. (%)						
Socioeconomic risks	248 (3.0%)	7,854 (2.2%)	0.05	122 (2.3%)	127 (2.4%)	0.006
Nicotine dependence	755 (9.0%)	20,690 (5.7%)	0.127	413 (7.8%)	419 (7.9%)	0.004
Hypertensive diseases	4,297 (51.3%)	31,472 (8.7%)	1.051	2,137 (40.2%)	2,286 (43.0%)	0.057
T2DM	4,441 (53.0%)	4,705 (1.3%)	1.429	1,819 (34.2%)	1,766 (33.2%)	0.021
T2DM with complications	2,551 (30.5%)	1,354 (0.4%)	0.916	808 (15.2%)	691 (13.0%)	0.063
Cerebrovascular diseases	161 (1.9%)	2,507 (0.7%)	0.109	96 (1.8%)	110 (2.1%)	0.019
Liver diseases	896 (10.7%)	5,077 (1.4%)	0.398	424 (8.0%)	446 (8.4%)	0.015
Hyperlipidemia	2,279 (27.2%)	10,696 (3.0%)	0.721	1,012 (19.0%)	1,039 (19.5%)	0.013
Acute MI	68 (0.8%)	671 (0.2%)	0.089	41 (0.8%)	41 (0.8%)	<0.001
Heart failure	306 (3.7%)	2,215 (0.6%)	0.212	175 (3.3%)	192 (3.6%)	0.018
Atherosclerosis	58 (0.7%)	487 (0.1%)	0.087	39 (0.7%)	50 (0.9%)	0.023
Peripheral vascular disease	79 (0.9%)	507 (0.1%)	0.11	41 (0.8%)	53 (1.0%)	0.024
CKD	344 (4.1%)	3,447 (1.0%)	0.202	226 (4.2%)	268 (5.0%)	0.038
Gastric ulcer	34 (0.4%)	824 (0.2%)	0.032	18 (0.3%)	21 (0.4%)	0.009
Connective tissue disorders	164 (2.0%)	3,873 (1.1%)	0.073	103 (1.9%)	101 (1.9%)	0.003
HIV disease	40 (0.5%)	940 (0.3%)	0.036	25 (0.5%)	26 (0.5%)	0.003

© 2026 Yen T et al. *JAMA Network Open*.

Chronic lower respiratory diseases	1,893 (22.6%)	28,882 (8.0%)	0.415	1,021 (19.2%)	1,095 (20.6%)	0.035
Pregnancy	118 (1.4%)	28,241 (7.8%)	0.308	93 (1.7%)	106 (2.0%)	0.018
Service types, No. (%)						
Office/ Outpatient Services	6,353 (75.8%)	173,229 (47.8%)	0.603	3,776 (71.0%)	3,849 (72.4%)	0.03
Preventive Medicine Services	2,441 (29.1%)	70,585 (19.5%)	0.227	1,518 (28.5%)	1,520 (28.6%)	0.001
ED Services	2,237 (26.7%)	60,173 (16.6%)	0.247	1,278 (24.0%)	1,307 (24.6%)	0.013
Hospital Inpatient Services	626 (7.5%)	18,620 (5.1%)	0.096	376 (7.1%)	432 (8.1%)	0.04
Medications, No. (%)						
Insulins	2,089 (24.9%)	4,946 (1.4%)	0.744	894 (16.8%)	928 (17.5%)	0.017
Sulfonylureas	1,102 (13.2%)	611 (611)	0.539	270 (5.1%)	200 (3.8%)	0.064
DPP-4 inhibitors	646 (7.7%)	119 (0.0%)	0.406	128 (2.4%)	84 (1.6%)	0.059
SGLT2 inhibitors	603 (7.2%)	75 (0.0%)	0.392	99 (1.9%)	49 (0.9%)	0.08
Thiazolidinediones	170 (2.0%)	44 (0.0%)	0.202	39 (0.7%)	24 (0.5%)	0.037
Alpha glucosidase inhibitors	23 (0.3%)	48 (0.0%)	0.069	11 (0.2%)	11 (0.2%)	<0.001
Estrogens	821 (9.8%)	48,601 (13.4%)	0.113	569 (10.7%)	577 (10.8%)	0.005
Rivaroxaban	89 (1.1%)	1,309 (0.4%)	0.084	61 (1.1%)	59 (1.1%)	0.004
Apixaban	111 (1.3%)	1,379 (0.4%)	0.103	76 (1.4%)	80 (1.5%)	0.006
Warfarin	110 (1.3%)	1,882 (0.5%)	0.083	68 (1.3%)	73 (1.4%)	0.008
Aspirin	857 (10.2%)	11,274 (3.1%)	0.288	454 (8.5%)	481 (9.0%)	0.018
Clopidogrel	97 (1.2%)	786 (0.2%)	0.114	58 (1.1%)	77 (1.4%)	0.032
Ticagrelor	23 (0.3%)	114 (0.0%)	0.062	12 (0.2%)	13 (0.2%)	0.004
Enoxaparin	547 (6.5%)	8,292 (2.3%)	0.208	316 (5.9%)	348 (6.5%)	0.025
BMI, mean (SD), kg/m^2	41.8 (9.4)	29.8 (8.3)	1.354	41.4 (9.5)	40.6 (9.2)	0.088
< 18.5, No. (%)	65 (0.8%)	7,111 (2.0%)	0.102	36 (0.7%)	31 (0.6%)	0.012
18.5 – 25, No. (%)	129 (1.5%)	70,707 (19.5%)	0.612	101 (1.9%)	89 (1.7%)	0.017
25 – 30, No. (%)	628 (7.5%)	67,974 (18.7%)	0.338	438 (8.2%)	403 (7.6%)	0.024
30 – 40, No. (%)	2,836 (33.9%)	67,661 (18.7%)	0.351	1,723 (32.4%)	1,771 (33.3%)	0.019
≥ 40, No. (%)	3,547 (42.3%)	26,328 (7.3%)	0.889	1,970 (37.0%)	2,206 (41.5%)	0.091
HbA1c, mean (SD), %	7.8 (2.3)	5.5 (1.1)	1.302	7.1 (2.1)	6.5 (1.8)	0.289
< 5.7, No. (%)	1,085 (13.0%)	30,132 (8.3%)	0.151	967 (18.2%)	1,210 (22.8%)	0.113
5.70 - 6.5, No. (%)	1,777 (21.2%)	8,409 (2.3%)	0.613	1,157 (21.8%)	1,400 (26.3%)	0.107
≥ 6.5, No. (%)	4,227 (50.5%)	2,655 (0.7%)	1.387	1,478 (27.8%)	1,316 (24.7%)	0.069

Abbreviations: BMI, body mass index; CKD, Chronic kidney disease; DPP-4, Dipeptidyl peptidase 4; ED, emergency department; GLP-1RA, glucagon-like peptide 1 receptor agonist; HbA1c, glycosylated hemoglobin; HIV, Human immunodeficiency virus; MI,

© 2026 Yen T et al. *JAMA Network Open*.

myocardial infarction; P, progestins; PSM, propensity score matching; SD, standard deviation; SERM, selective estrogen receptor modulators; SGLT2, Sodium-glucose co-transporter 2; SMD, standardized mean difference; T2DM, Type 2 diabetes mellitus. [a], SMD less than 0.10 indicates that the two comparison groups were well balanced. Native American refers to American Indian or Alaska Native; Pacific Islander refers to Native Hawaiian or Other Pacific Islander. In accordance with the HIPAA Privacy Rule, covariates with fewer than 10 patients were not presented in this table, including dementia and the use of SERM, dabigatran, edoxaban, and prasugrel.

© 2026 Yen T et al. *JAMA Network Open*.
