# Supplementary material for: GLP-1 Receptor Agonists Plus Progestins and Endometrial Cancer Risk in Nonmalignant Uterine Diseases
Source: JAMA Netw Open. 2026 Feb 10;9(2):e2558205. doi: 10.1001/jamanetworkopen.2025.58205 (PMC12892152; doi:10.1001/jamanetworkopen.2025.58205)
Supplement: Supplement 2. — Data Sharing Statement [file jamanetwopen-e2558205-s002.pdf]

Data Sharing Statement

Yen. GLP-1 Receptor Agonists Plus Progestins on Endometrial Cancer Risk in Nonmalignant Uterine Diseases. *JAMA Netw Open*. Published February 10, 2026.
doi:10.1001/jamanetworkopen.2025.58205

Data
Data available: No

Additional Information
Explanation for why data not available: Due to the proprietary nature of the data provided by TriNetX, the dataset cannot be made publicly available. However, the data supporting the findings of this study can be accessed through TriNetX, subject to their data licensing agreement. Interested researchers may contact TriNetX directly at join@trinetx.com to request access.
